# Administration of *Porphyromonas gingivalis* in pregnant mice enhances glycolysis and histone lactylation/ADAM17 leading to cleft palate in offspring

**DOI:** 10.1038/s41368-025-00347-x

**Published:** 2025-03-13

**Authors:** Xige Zhao, Xiaoyu Zheng, Yijia Wang, Jing Chen, Xiaotong Wang, Xia Peng, Dong Yuan, Ying Liu, Zhiwei Wang, Juan Du

**Affiliations:** 1 Laboratory of Orofacial Development, Laboratory of Molecular Signaling and Stem Cells Therapy, Molecular Laboratory for Gene Therapy and Tooth Regeneration, Beijing Key Laboratory of Tooth Regeneration and Function Reconstruction, Capital Medical University School of Stomatology, Beijing, China; 2 Department of geriatric dentistry, Capital Medical University School of Stomatology, Beijing, China

**Keywords:** Differentiation, Periodontitis, Risk factors, Entosis, Mechanisms of disease

## Abstract

Periodontal disease is a risk factor for many systemic diseases such as Alzheimer’s disease and adverse pregnancy outcomes. Cleft palate (CP), the most common congenital craniofacial defect, has a multifaceted etiology influenced by complex genetic and environmental risk factors such as maternal bacterial or virus infection. A prior case-control study revealed a surprisingly strong association between maternal periodontal disease and CP in offspring. However, the precise relationship remains unclear. In this study, the relationship between maternal oral pathogen and CP in offspring was studied by sonicated *P. gingivalis* injected intravenously and orally into pregnant mice. We investigated an obvious increasing CP (12.5%) in sonicated *P. gingivalis* group which had inhibited osteogenesis in mesenchyme and blocked efferocytosis in epithelium. Then glycolysis and H4K12 lactylation (H4K12la) were detected to elevate in both mouse embryonic palatal mesenchyme (MEPM) cells and macrophages under *P. gingivalis* exposure which further promoted the transcription of metallopeptidase domain17 (ADAM17), subsequently mediated the shedding of transforming growth factor-beta receptor 1 (TGFBR1) in MEPM cells and mer tyrosine kinase (MerTK) in macrophages and resulted in the suppression of efferocytosis and osteogenesis in palate, eventually caused abnormalities in palate fusion and ossification. The abnormal efferocytosis also led to a predominance of M1 macrophages, which indirectly inhibited palatal osteogenesis via extracellular vesicles. Furthermore, pharmacological ADAM17 inhibition could ameliorate the abnormality of *P. gingivalis*-induced abnormal palate development. Therefore, our study extends the knowledge of how maternal oral pathogen affects fetal palate development and provides a novel perspective to understand the pathogenesis of CP.

## Introduction

Cleft palate (CP), the most common congenital craniofacial defect, can significantly impact quality of life, even following surgical interventions, and places a considerable strain on families and society.^1^ CP has a multifaceted etiology influenced by complex genetic and environmental risk factors.^2^ Maternal environment is important to embryonic development, and disturbing it may cause deformity. The maternal intrauterine microenvironment, which surrounds the fetus and affects it directly, was once believed to be sterile. However, recent evidences have demonstrated the vertical transmission of maternal oral microbes to the uterus, potentially introducing them into the intrauterine microenvironment.^3,4^ Periodontitis, one of the major public health issues often caused by *Porphyromonas gingivalis* (*P. gingivalis*) among other periodontal pathogens, is confirmed relating to more and more systemic diseases such as Alzheimer’s disease, atherosclerosis and adverse pregnancy outcomes.^5^ An unexpectedly strong association between maternal periodontal disease and CP in children was observed in a previous case-control study.^6^ However, the exact mechanism of this relationship remains unclear.

Mice is the most common animal model in exploring etiology of CP. The secondary palatal shelves in mice, between embryonic days (E) 12.5 and 15.5, undergo a sequence of vertical growth, elevation, horizontal growth, fusion, and osteogenesis.^7^ This process mirrors human palate development to a large extent and any disturbance during the process will result in CP.^7^ Recently, metabolic shifts stemming from environmental and dietary changes has been revealed to influence cell fate in both physiological and pathological states via epigenetic effect.^8^ Studies have observed dramatic shifts in glucose metabolism during the migration and differentiation of neural crest cells, which share homology with palatine mesenchymal cells.^9,10^ Our previous study also demonstrated that mouse embryonic palatal mesenchyme (MEPM) cells at E13.5 undergo significant growth and primarily depend on glycolysis, then shift to oxidative phosphorylation (OXPHOS) as they adapt to osteogenesis in response to functional demands and environmental cues at E15.5.^11^ However, little is known about whether aberrated shifts in glucose metabolism involve in the increasing risk of CP. On the other hand, numerous adverse outcomes in offspring, including metabolic disorders, have been associated with maternal *P. gingivalis* infection.^12,13^ Moreover, prior studies suggested that *P. gingivalis* infection can trigger a metabolic shift from OXPHOS to glycolysis as an adaptation to stressful conditions,^14,15^ which is opposite to the normal metabolic progression of palate development in our previous study.^11^ We then wonder if *P. gingivalis* infection disrupts glucose metabolism will affect palatogenesis.

Historically, studies on palate development have focused predominantly on functional changes in MEPM cells, while potential contributions of immune cells have been largely overlooked. Emerging evidences indicate that macrophages (Mφs) play an active role during the key stage of palatal fusion by phagocytosing apoptotic epithelial fragments in the decomposed midpalate epithelial suture (MES).^16,17^ This activity fine-tunes palatal fusion, suggesting a significant role for Mφ-mediated efferocytosis in palatal fusion regulation. It has also been demonstrated that sustained glycolysis can negatively impact on Mφ functions. For example, the functions such as phagocytosis and chemotaxis of microglia, the resident Mφs in brain, can be compromised when glycolytic metabolism is activated.^18^ Nevertheless, the relationship between Mφ and metabolism in palate development has not been reported.

While the regulatory role of metabolic pathways in cell function has garnered considerable attention, the specific mechanisms involved remain largely elusive. Recently, a variety of histone modification marks derived from cellular metabolites have been discovered.^19^ Among these, lactate, a product of glycolysis, has been identified as a substrate for histone lactylation. Histone lactylation has been shown to regulate cell functions, such as Mφ polarization and somatic cell reprogramming.^20,21^ Additionally, our previous study suggested that changes in the maternal gut microbiome could affect fetal palatal development, potentially due to metabolic disorders and lactate accumulation,^22^ which meanest histone lactylation might participate in the process.

In this study, we demonstrated that *P. gingivalis* exposure caused CP in mice which elevates glycolysis and subsequent histone lactylation, esp. H4K12 lactylation in both Mφs and MEPM cells. This increased lactylation promoted the transcription of metallopeptidase domain17 (ADAM17) which mediated the shedding of Mer tyrosine kinase (MerTK) in Mφs and transforming growth factor-beta receptor 1 (TGFBR1) in MEPM cells, inducing the suppression of Mφ efferocytosis in epithelium and MEPM cells osteogenesis, respectively, and ultimately resulting in abnormalities in palate shelves fusion and ossification. Blocking ADAM17 or histone lactylation could rescue the occurrence of CP. Furthermore, the abnormal efferocytosis also led to a predominance of M1 Mφs, which indirectly inhibited palatal osteogenesis via small extracellular vesicles (sEVs). Therefore, our study describes a cascade process from an initial perturbation of how maternal oral pathogen impact on fetal palate development, highlights novel aspects on etiology of congenital malformations and its potential interceptive therapy.

## Results

### *P. gingivalis* induced CP with abnormal palate shelves fusion and osteogenesis in palate development in vivo

**Fig. 1:**
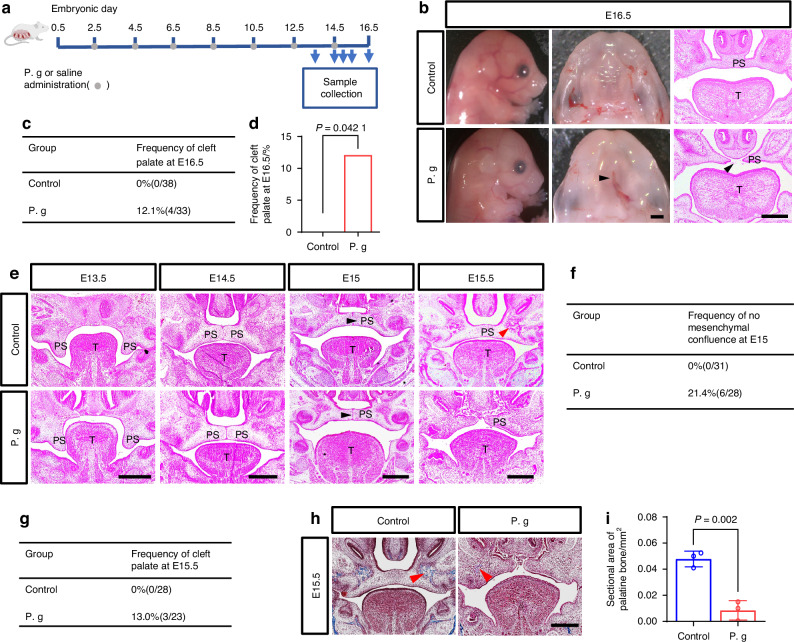
In vivo evaluation of palatal phenotypes under sonicated *P. gingivalis* treatment. **a** Experimental design and time schedule of the study. **b** Lateral and occlusal views and histological observation of mice palates after treatment with or without sonicated *P. gingivalis* (P. g). The arrowheads indicate the cleft. Bar, 200 μm. PS represents palatal shelves and T represents togue. **c**, **d** Frequency of cleft palate in P. g group compared with Control group, *P* value was calculated by Fisher’s exact test. **e** Sequential histological sections from E13.5 to E15.5, black arrows indicate the MES, red arrows indicate osteogenic center. Bar, 200 μm. The frequency of no mesenchymal confluence at E15 (**f**) and the frequency of cleft in control and P. g mice at E15.5 (**g**). **h**, **i** Masson staining and statistical analysis (*n* = 3). red arrows indicate osteogenic center. Bar, 200 μm. The data are shown as the mean ± SD and were statistically analyzed by two-tailed Student’s *t*-test (**h**)

Sonicated *P. gingivalis* is a common method in investigating effects of *P. gingivalis* on the body.^23^ To examine the potential translocation of sonicated *P. gingivalis* to the fetal palate and to evaluate the temporal variation in its concentration during the critical period of palatal development, we firstly constructed a mouse model of experimental *P. gingivalis* exposure during pregnant (Fig. 1a),^24^ and assessed the presence of *P. gingivalis* gingipain R1 (RgpA) in the amniotic fluid and fetal palatal process utilizing indirect Enzyme-Linked ImmunoSorbent Assay (ELISA). As depicted in Supplementary Fig. S1a, b, the injection of sonicated *P. gingivalis* resulted in a significant elevation of RgpA levels in the amniotic fluid and fetal palate tissue, with a progressive increase observed between E13.5 and E15.5. We then evaluated the impact of sonicated *P. gingivalis* exposure on palate development. In the sonicated *P. gingivalis*-treated group, CP (12.1%, 4 out of 33 embryos) was evident at E16.5, revealed by direct observation under a stereoscope and in histological sections stained with hematoxylin and eosin (HE) (Fig. 1b–d, Supplementary Table 1, Supplementary Table 2).

To better understand the moment of cleft origin and the persistence of the phenotypes, we further analyzed sequential histological sections from E13.5 to E15.5 (Fig. 1e). At E13.5, the palatal shelves grew vertically adjacent to the tongue. By E14.5, the secondary palatal shelves started to meet at the middle of the palate. At this point, no abnormal palatal phenotypes were observed under *P. gingivalis* exposure. However, at E15.0, unlike in the control palates when the midline epithelial remnants started to degrade and partial mesenchymal confluence was achieved, the epithelial remnants in the *P. gingivalis*-treated group were still present at the midline without any mesenchymal confluence (Fig. 1e, f). By E15.5, while complete fusion of the secondary palatal shelves had occurred in the control palate, the fusion failed in the *P. gingivalis*-treated group with scattered epithelial cell fragments at the interface (Fig. 1e, g). It appeared that the palatal shelves, which made contact at E15.0, became detached by E15.5.

Moreover, HE staining results indicated a distinct osteogenic center in the E15.5 control palate, which was absent in the *P. gingivalis*-treated palate (Fig. 1e). To investigate whether *P. gingivalis* exposure affected palate bone formation later in development, we compared the bone area of the palate using Masson staining. Both Masson staining and statistical analysis revealed a significant decrease in bone areas in the E15.5 *P. gingivalis*-treated palate compared with the control (Fig. 1h, i).

In summary, no morphological differences were observed in the *P. gingivalis*-treated mice by E14.5. However, disruption of the epithelial fusion began around E15.0, and the palatal phenotypes became evident from E15.5 onwards with disturbed palate shelves fusion and osteogenesis.

### MEPM cells migration and osteogenesis were inhibited under *P. gingivalis* exposure

**Fig. 2:**
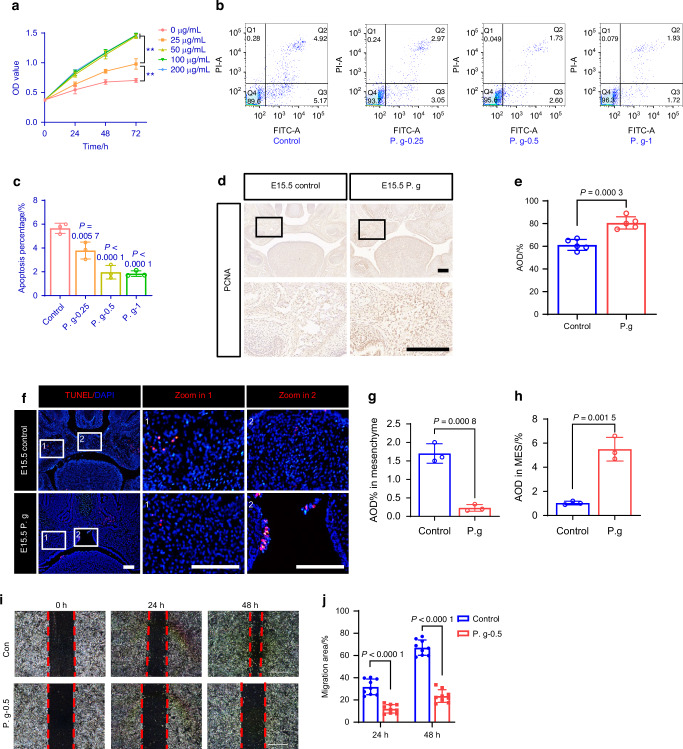
Exposure to *P. gingivalis* (P. g) facilitated the proliferation of MEPM cells and inhibited their apoptosis. **a** The growth curve of MEPM cells in P. g of different concentrations (*n* = 6, **P < 0.01). **b** AnnexinV-FITC/PI flow cytometric analysis and **c** statistical data of MEPM cell apoptosis (*n* = 3). **d**, **e** PCNA IHC in E15.5 fetal palate from mice treated with or without P. g, with quantification of positive cells (right panel, *n* = 5). Bar, 100 μm. **f**, **g**, **h** TUNEL assay in E15.5 fetal palate from mice treated with or without P. g, with quantification of positive cells (right panel, *n* = 3). Bar, 100 μm. **i** Scratch assay for cell migration and cell migration rate was quantified by calculating the wound area (**j**) (*n* = 9). The data are shown as the mean ± SD and were statistically analyzed by one-way ANOVA with Tukey’s multiple-comparison test (**a**, **c**, **j**) or two-tailed Student’s *t*-test (**e**, **g**, **h**). All the *P* values were two-sided and adjustments were made for multiple comparisons

MEPM cells migrate to the osteogenic region and undergo proliferation and differentiation to form the bone structures of the palate. To explore the mechanism of the abnormal osteogenesis induced by sonicated *P. gingivalis*, we further investigated its impact on the biological behavior of MEPM cells in vitro and in vivo. Using a Cell Counting Kit-8 (CCK-8) assay, we observed that sonicated *P. gingivalis* (at concentrations of 0, 0.25, 0.5, and 1 μg/mL) stimulated MEPM cell proliferation, with the magnitude of the effect corresponding to the concentration and a significant effect was achieved at a concentration of 0.5 μg/mL (Fig. 2a). Flow cytometry analyses further showed that sonicated *P. gingivalis* inhibited MEPM cell apoptosis (Fig. 2b, c). Next, proliferating cell nuclear antigen (PCNA) immunohistochemical staining was utilized to evaluate the proliferation of MEPM cells in vivo. Consistent with in vitro findings, our results revealed an upregulation of PCNA expression in the palatal mesenchyme following treatment with sonicated *P. gingivalis* (Fig. 2d, e). Additionally, the Terminal deoxynucleotidyl transferase dUTP nick end labeling (TUNEL) assay was also employed to detect apoptotic cells. We observed a reduction in apoptotic cells within the *P. gingivalis*-exposed mesenchyme (Fig. 2f, g), whereas an increase in epithelial apoptosis was observed around the MES (Fig. 2f, h). These results suggest that osteogenic disorders in mesenchyme caused by sonicated *P. gingivalis* were not due to a deficiency in osteoprogenitors.

**Fig. 3:**
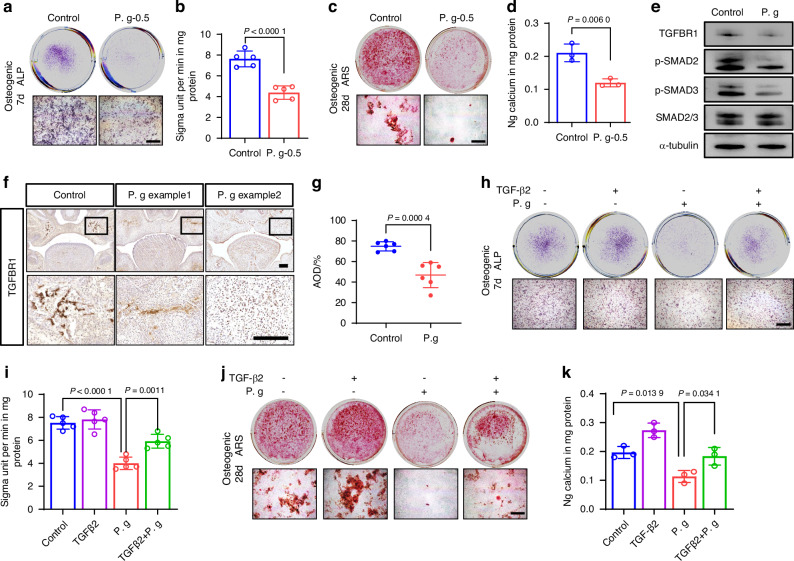
Sonicated *P. gingivalis* (P. g) induced osteogenic inhibition is related to decreased TGFBR1 in MEPM cells. **a**, **b** The expression levels of ALP were detected using ALP staining and ALP assay (*n* = 5). **c** Alizarin red S staining on 28 days and **d** the calcium concentration was determined by measuring the absorbance at 562 nm on a multiplate reader as shown in the bottom panel (*n* = 3). Bar, 500 μm. **e** Western blot assay of the protein in classical TGFβ pathway. **f**, **g** TGFBR1 IHC in fetal palate from mice treated with or without P. g, with quantification of positive cells (right panel, *n* = 6). Bar, 100 μm. **h**, **i** The expression levels of ALP were detected using ALP staining and ALP assay (*n* = 5). Bar, 500 μm. **j** Alizarin red S staining on 28 days and **k** the calcium concentration was determined by measuring the absorbance at 562 nm on a multiplate reader (*n* = 3). Bar, 500 μm. The data are shown as the mean ± SD and were statistically analyzed by one-way ANOVA with Tukey’s multiple-comparison test (**i**, **k**) or two-tailed Student’s *t*-test (**b**, **d**, **g**). All the *P* values were two-sided and adjustments were made for multiple comparisons

We then used the concentration of 0.5 μg/mL in subsequent in vitro experiments. Scratch assay results revealed that *P. gingivalis* inhibited MEPM cells migration (Fig. 2I, j). Both Alkaline phosphatase (ALP) staining (Fig. 3a, b), an early marker of osteogenesis and alizarin red staining (ARS) (Fig. 3c, d), the late osteogenic indicator displayed blocked osteogenic differentiation under sonicated *P. gingivalis* exposure after MEPM cells osteogenic induction for 7 and 28 days, along with a decreased osteogenesis indicators such as osteocalcin (*Ocn*), osterix (*Osx*), *Alp*, runt related transcription factor 2 (*Runx2*) (Supplementary Fig. S2). These data suggest that *P. gingivalis* inhibited the osteogenic capacity of MEPM cells, consistent with in vivo phenotypes.

### Osteogenic inhibition induced by *P. gingivalis* is related to decreased TGFBR1

Given our prior confirmation of TGFBR1’s crucial role in palatal osteogenic differentiation,^25,26^ we focused on mediators closely associated with the classical TGFβ pathway. We found that the protein levels of TGFBR1, p- Suppressor of mother against decapentaplegic 2 (p-SMAD2), and p-SMAD3 were significantly downregulated upon MEPM cells exposure to sonicated *P. gingivalis* (Fig. 3e, Supplementary Fig. S3a). This downregulation of TGFBR1, particularly in regions of palatal osteogenesis, was further confirmed in vivo using immunohistochemistry (Fig. 3f, g).

To further illustrate TGFBR1’s association with the abnormal osteogenesis caused by sonicated *P. gingivalis*, we introduced TGFβ2 in vitro to activate TGFBR1 because TGFβ2 is mainly expressed in the palatal mesenchyme.^27^ ALP assay and ARS showed that the addition of 10 ng/mL TGFβ2 could partially reverse the inhibition of osteogenesis induced by sonicated *P. gingivalis* (Fig. 3h–k). This indicates that the abnormal osteogenic differentiation induced by sonicated *P. gingivalis* is associated with the TGF-β pathway through TGFBR1.

### Sonicated *P. gingivalis* induced TGFBR1 cleavage in MEPM cells by upregulating H4K12la/ADAM17

**Fig. 4:**
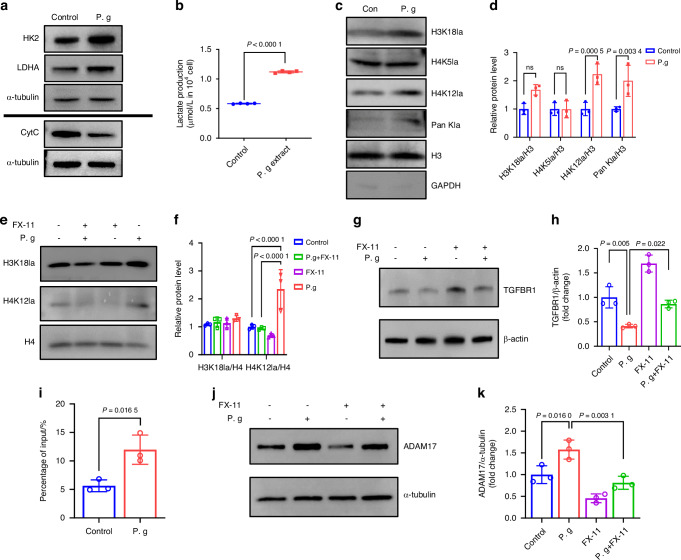
Sonicated *P. gingivalis* (P. g) induced TGFBR1 cleavage in MEPM cells by upregulating H4K12la/ADAM17. **a** Western blot assay of the markers in glycolysis and OXPHOs in MEPM cells treated with or without P. g. **b** The production of lactate (*n* = 4). **c** Western blot analysis of Pan- and site-specific histone lactylation, with quantification of protein levels as shown in (**d**) (*n* = 3). **e** Western blot analysis of H4K12la and H3K18la after added P. g and (or) FX-11, with quantification of protein levels as shown in (**f**) (*n* = 3). **g** Western blot analysis of TGFBR1, with quantification of protein levels as shown in (**h**) (*n* = 3). **i** ChIP-qPCR analysis of the ADAM17 promoters was performed using antibodies against H4K12la in MEPM cells (*n* = 3). **j** Western blot analysis of ADAM17 after added P. g and (or) FX-11, with quantification of protein levels as shown in (**k**) (*n* = 3). The data are shown as the mean ± SD and were statistically analyzed by one-way ANOVA with Tukey’s multiple-comparison test (**d**, **f**, **h**, **k**) or two-tailed Student’s *t*-test (**b**, **i**). All the *P* values were two-sided and adjustments were made for multiple comparisons

Recently glucose metabolic reprogramming was reported important during embryonic development esp. in neural crest.^9,10^ To investigate if the reduction in TGFBR1 was related to glucose metabolic reprogramming, we first evaluated the key mediators of glycolysis and OXPHOS. Western blot analysis revealed that hexokinase-2 (HK2) and lactate dehydrogenase A (LDHA), markers of glycolysis, were upregulated after treatment with sonicated *P. gingivalis*. In contrast, cytochrome c (CytC), a marker of OXPHOS, was downregulated following sonicated *P. gingivalis* treatment (Fig. 4a, Supplementary Fig. S3b). Additionally, the lactate level was significantly increased in the sonicated *P. gingivalis* group (Fig. 4b). These results demonstrated that *P. gingivalis* reversed metabolic reprogramming from OXPHOS to glycolysis in MEPM cells.

Currently histone lactylation induced by lactate is a key epigenetic regulation,^20^ we hypothesized that histone lactylation might be altered under sonicated *P. gingivalis* exposure which was confirmed by western blot analysis with an increase of histone lactylation, specifically H4K12la and H3K18la (Fig. 4c, d).

Next, we explored the biological function of histone lactylation induced by sonicated *P. gingivalis* in MEPM cells. We adjusted the level of histone lactylation in MEPM cells by treating them with sonicated *P. gingivalis* and/or the lactate dehydrogenase inhibitor, FX-11. As expected, the elevated level of H4K12la caused by sonicated *P. gingivalis* was effectively inhibited by 9 μmol/L FX-11, while changes in H3K18la were not significant (Fig. 4e, f). We then focused on H4K12la in subsequent experiments. Importantly, the inhibition of H4K12la could rescue the downregulation of TGFBR1 induced by sonicated *P. gingivalis*, suggesting a potential causal link between H4K12la and TGFBR1 (Fig. 4g, h).

To determine whether a direct relationship exists between these factors, we examined MEPM cells with ChIP-qPCR using an antibody against H4K12la. The promoter of *Tgfbr1* was not detected after treatment with the anti-H4K12la antibody, indicating there might not be a direct interaction between them (Supplementary Fig. S4).

Given that ADAM17 has been previously reported as a protease that cleaves the extracellular region of TGFBR1, and it might be activated in inflammatory or high-glucose environments.^28,29^ Consequently, we aimed to examine the link between histone lactylation and ADAM17 to determine if ADAM17 mediated the indirect regulation of TGFBR1 by H4K12la. Indeed, ChIP-qPCR demonstrated an increase in H4K12la enrichment in the promoter region of *Adam17* (Fig. 4i). Consistently, western blot confirmed the upregulation of ADAM17 expression after sonicated *P. gingivalis* treatment (Fig. 4j, k). Collectively, these results indicate that H4K12la modification activates the transcription of *Adam17* in MEPM cells.

**Fig. 5:**
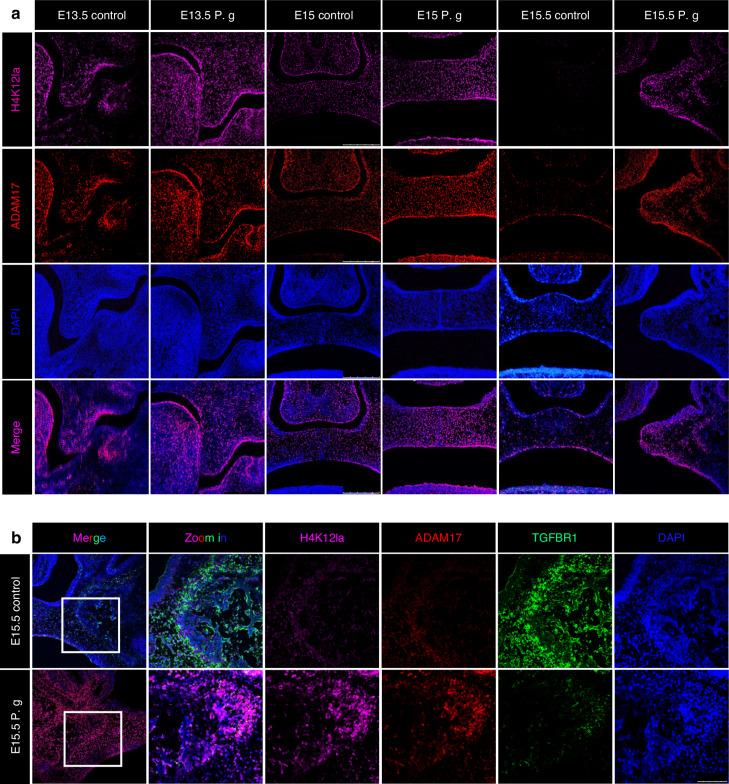
Co-staining of H4K12la/ADAM17/TGFBR1 in palate tissue. **a** The distribution and intensity of H4K12la and ADAM17 during palate development. Bar, 250 μm. **b** Co-staining of H4K12la/ADAM17/TGFBR1 at E15.5. P. g represents *P. gingivalis.* Bar, 100 μm

Lastly, we further investigated the distribution and intensity of H4K12la, ADAM17, and TGFBR1 during palate development in vivo. Consistent with our in vitro results, in vivo immunofluorescence findings showed that H4K12la and ADAM17 had similar distributions, with a substantial amount of co-localization (Fig. 5a). The signal intensity of H4K12la and ADAM17 was significantly increased in mice treated with sonicated *P. gingivalis* (Fig. 5a, Supplementary Fig. S5a, b). Furthermore, a time-course analysis revealed that the signal intensities of H4K12la and ADAM17 gradually decreased with embryonic development in normal mice, whereas these markers remained at a consistently high level in mice treated with sonicated *P. gingivalis* (Supplementary Fig. S5a, b).

Specifically, at the primary osteogenic site during the osteogenic stage (E15.5), we observed that TGFBR1-positive cells expressed lower levels of H4K12la and ADAM17 in the normal palate. In contrast, in the sonicated *P. gingivalis*-treated CP, the number of TGFBR1-positive cells significantly decreased due to the continuous expression of H4K12la and ADAM17 (Fig. 5b).

These findings suggest that the increase in H4K12la induced by sonicated *P. gingivalis* promotes the expression of ADAM17 which further cleaves TGFBR1, thereby inhibiting osteogenic differentiation in MEPM cells and inducing abnormal palate development.

### Decreased MerTK in Mφs under sonicated *P.gingivalis* exposure caused abnormal palate shelves fusion

**Fig. 6:**
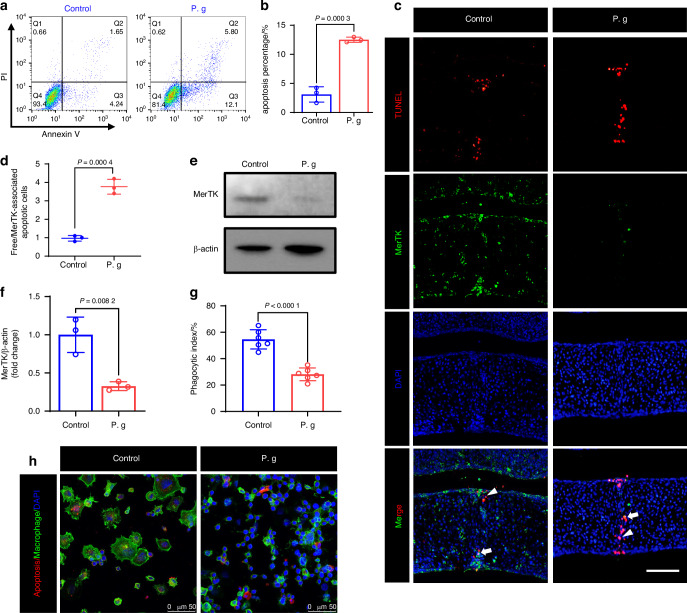
Sonicated *P. gingivalis* (P. g) induced abnormal fusion is related to decreased MerTK in macrophages. **a**, **b** AnnexinV-FITC/PI flow cytometric analysis and statistical data of MEE cell apoptosis (*n* = 3). **c** Co-staining of MerTK positive macrophage (green) in TUNEL-positive regions (red), with **d** quantification of Free/MerTK-associated apoptotic cells (*n* = 3). Free apoptotic cells, white arrows. MerTK associated apoptotic cells, white arrowheads. Bar, 100 μm. **e** Western blot analysis of MerTK in Raw 264.7 macrophage, with quantification of protein levels in (**f**) (*n* = 3). **g**, **h** Representative fluorescent images showing engulfing of apoptotic MEE cells by Raw 264.7 macrophages in vitro and phagocytic index based on the fluorescent image (*n* = 6). The data are shown as the mean ± SD and were statistically analyzed by two-tailed Student’s *t*-test

In addition to osteogenic abnormalities, as previously mentioned, fetal mice treated with sonicated *P. gingivalis* also displayed persistent epithelial remnants at the fusion stage which is usually medial edge epithelial (MEE) cells, leading to abnormal palatal fusion (Fig. 1e). To investigate the reason of the abnormity, we then evaluated the effect of sonicated *P. gingivalis* on the apoptosis of MEE. Using flow cytometry analysis, we observed that sonicated *P. gingivalis* promoted apoptosis in MEE cells (Fig. 6a, b). This suggests that the abnormal fusion induced by sonicated *P. gingivalis* may not be due to a reduction in MEE apoptosis.

Subsequently, we performed immunofluorescence staining of tissue sections at E15 to examine apoptotic MEE in vivo. TUNEL assay revealed clear apoptosis signals in the midline epithelium of palate shelves in both the control and sonicated *P. gingivalis*-treated groups. However, in the control group, apoptotic cells were predominantly located in MerTK-positive areas, whereas the ratio of free to MerTK-associated apoptotic cells significantly increased in the sonicated *P. gingivalis*-treated group (Fig. 6c, d). As MerTK plays a crucial role in binding molecules on the apoptotic cell surface or binding bridging molecules that interact with the apoptotic cell surface during efferocytosis, these results imply that sonicated *P. gingivalis* may induce inefficient efferocytosis by decreasing MerTK expression in Mφs.

We further investigated whether the expression of the MerTK in Raw 264.7 and isolated mice Bone marrow derived-macrophages (BMDM) was affected by sonicated *P. gingivalis* in vitro. As shown in Fig. 6e, f and Supplementary Fig. S6a, MerTK expression decreased when exposed to sonicated *P. gingivalis*. Given that BMDM cells are not amenable to passage and are unsuitable for extended culture periods, we predominantly selected Raw264.7 macrophages for subsequent experimental procedures.^30^ We then explored the potential impact of sonicated *P. gingivalis* on Mφ efferocytosis in vitro. After the addition of apoptotic cells, Raw 264.7 cells in the control group displayed an enlarged cellular surface area along with the formation of additional filopodia-like structures, which might act as phagocytic tentacles to facilitate phagocytosis. Conversely, in the sonicated *P. gingivalis* group, Raw 264.7 cells exhibited a dystrophic morphology, and the phagocytic index, indicating Mφ efferocytosis efficiency, was significantly decreased (Fig. 6g, h). Therefore, our data indicate that the abnormal palatal fusion induced by sonicated *P. gingivalis* may be related to decreasing MerTK which causes inefficient efferocytosis of Mφs.

### Sonicated *P. gingivalis* induced MerTK cleavage in Mφs through upregulating H4K12la/ADAM17

**Fig. 7:**
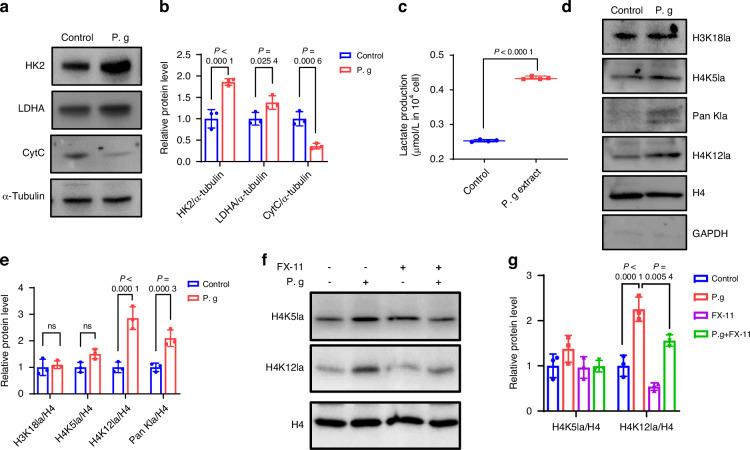
In macrophages, glycolysis and H4K12la were also observed to increase upon exposure to *P. gingivalis* (P. g). **a** Western blot assay of the markers in glycolysis and OXPHOs in macrophages treated with or without P. g, with quantification of protein levels as shown in (**b**) (*n* = 3). **c** The production of lactate (*n* = 4). **d** Western blot analysis of Pan- and site-specific histone lactylation, with quantification of protein levels as shown in (**e**) (*n* = 3). **f** Western blot analysis of H4K12la and H4K5la after added P. g and (or) FX-11, with (**g**) quantification of protein levels (*n* = 3). The data are shown as the mean ± SD and were statistically analyzed by one-way ANOVA with Tukey’s multiple-comparison test (**b**, **e**, **g**) or two-tailed Student’s *t*-test (**c**). All the *P* values were two-sided and adjustments were made for multiple comparisons

To investigate whether the decrease of MerTK was related to glucose metabolic reprogramming and histone lactylation, we identified the changes of glucose metabolism, lactate levels, and histone lactylation in Raw 264.7 cells after sonicated *P. gingivalis* treatment similar to the MEPM cells. Our results indicated an increase in the expressions of glycolytic mediators, HK2 and LDHA, a decrease in the expression of Cytc (OXPHOS mediator), and higher levels of lactate and histone lactylation in Raw 264.7 cells treated with sonicated *P. gingivalis*, especially H4K5la and H4K12la (Fig. 7a–e).

**Fig. 8:**
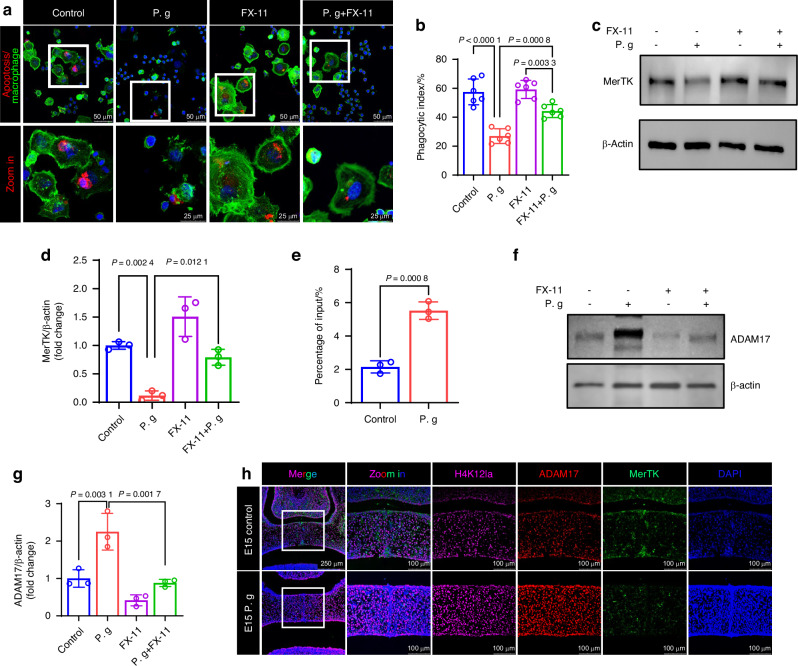
Sonicated *P. gingivalis* (P. g) induced MerTK cleavage in Raw 264.7 macrophages by upregulating H4K12la/ADAM17. **a** Representative fluorescent images showing engulfing of apoptotic MEE cells by macrophages and (**b**) phagocytic index based on the fluorescent image (*n* = 6). **c** Western blot analysis of MerTK, with (**d**) quantification of protein levels (n = 3). **e** ChIP-qPCR analysis of the ADAM17 promoters was performed using antibodies against H4K12la in macrophages (*n* = 3). **f** Western blot analysis of ADAM17 after added P. g and (or) FX-11, with (**g**) quantification of protein levels (*n* = 3). **h** Co-staining of H4K12la/ADAM17/MerTK. The data are shown as the mean ± SD and were statistically analyzed by one-way ANOVA with Tukey’s multiple-comparison test (**b**, **d**, **g**) or two-tailed Student’s *t*-test (**e**). All the *P* values were two-sided and adjustments were made for multiple comparisons

Subsequently, we introduced FX-11 to manipulate histone lactylation levels and examine the relationship between changes in histone lactylation and MerTK expression. The elevated level of H4K12la induced by sonicated *P. gingivalis* was effectively inhibited by 9 μmol/L FX-11 (Fig. 7f, g). Moreover, the suppression of H4K12la was able to rescue the downregulation of MerTK and efferocytosis instigated by sonicated *P. gingivalis* (Fig. 8a–d). This suggests that the decrease of MerTK in Raw 264.7 cells may also be related to H4K12la. Interestingly, previous report has indicated that MerTK is also susceptible to ADAM17 cleavage,^31^ which helps us speculate that ADAM17 may also mediate the indirect regulation of H4K12la on MerTK. Indeed, our ChIP-qPCR and western blot results confirmed our speculation, suggesting that the elevation of H4K12la induced by sonicated *P. gingivalis* promoted the expression of ADAM17 (Fig. 8e–g). This may further inhibit MerTK expression and thus suppress efferocytosis in Mφs. Additionally, treatment of isolated mice BMDM with sonicated *P. gingivalis* also resulted in increases in HAK12la and ADAM17 (Supplementary Fig. S6b, c).

Moreover, we analyzed the distribution and intensity of H4K12la/ADAM17/MerTK at the palate fusion stage (E15) in vivo. Immunofluorescence results showed that in the normal palate, despite a relatively high level of basal glycolysis at E15, a significant number of cells expressed H4K12la and ADAM17 while MerTK-positive Mφs recruited around the MES expressed lower levels of H4K12la and ADAM17. In contrast, in the sonicated *P. gingivalis* group, MerTK-positive cells were rarely distributed around the MES due to the high expression of H4K12la and ADAM17 (Fig. 8h).

### Mφs exposed to sonicated *P. gingivalis* cannot undergo a phenotypic switch upon efferocytosis during palate fusion

Previous studies have demonstrated that successful efferocytosis initiates signals for the resolution of inflammation, leading to a phenotypic switch from the inflammatory M1 phenotype to the anti-inflammatory M2 phenotype.^32^ As such, we hypothesized that during palate shelves fusion, Mφs would undergo a phenotypic switch after phagocytizing apoptotic MEE, while inefficient efferocytosis induced by sonicated *P. gingivalis* could result in a failed phenotypic switch. To test this hypothesis, we began with in vitro experiments. Before being co-cultured with DiI-labeled apoptotic MEE cells, the control Raw 264.7 cells were in an unpolarized state, while the sonicated *P. gingivalis*-pretreated Raw 264.7 cells showed M1 polarization. After a 30-min co-culture, the M1 phenotype was dominant in both groups. Two hours post-co-culture, Raw 264.7 cells in the control group switched to M2 as most of the apoptotic MEE cells were cleared. However, in the sonicated *P. gingivalis* group, only a few Raw 264.7 cells that recognized and phagocytized apoptotic MEE cells switched to M2 (Supplementary Fig. S7).

**Fig. 9:**
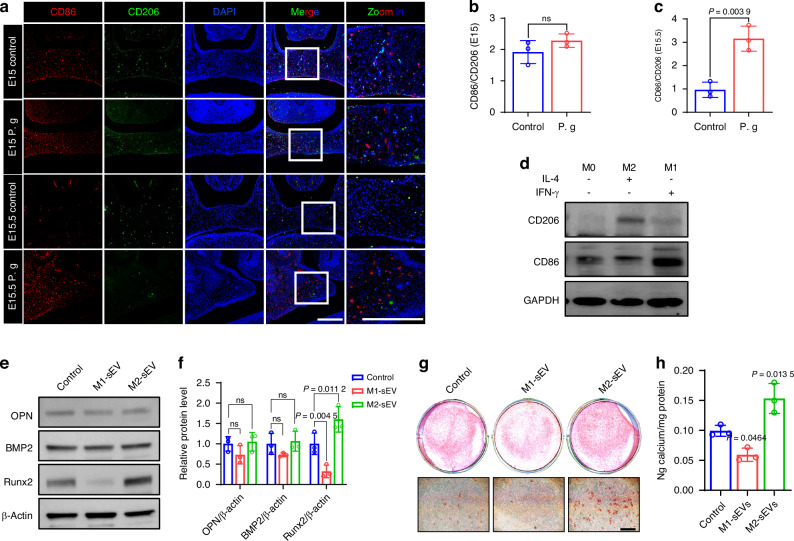
Macrophages exposed to sonicated *P. gingivalis* (P. g) cannot undergo a phenotypic switch upon efferocytosis during palate fusion, and M2-sEVs promoted MEPM osteogenesis while M1-sEVs inhibited it. **a** Co-staining of CD86 /CD206 and quantification of CD86/CD206 at **b** E15 or **c** E15.5. Bar, 200 μm (*n* = 3). **d** Macrophages were polarized into M1 or M2 types in vitro *a*nd western blot analysis of CD86 and CD206 for identification. **e** Western blot analysis of osteogenic protein in MEPM cells treated with M1-sEVs or M2-sEVs, with (**f**) quantification of protein levels (*n* = 3). **g** Alizarin red S staining on 21 days for MEPM cells treated with M1-sEVs or M2-sEVs and **h** the calcium concentration was determined by measuring the absorbance at 562 nm on a multiplate reader (*n* = 3). The data are shown as the mean ± SD and were statistically analyzed by one-way ANOVA with Tukey’s multiple-comparison test (**f**, **h**) or two-tailed Student’s *t*-test (**b**, **c**). All the *P* values were two-sided and adjustments were made for multiple comparisons

Subsequently, we observed Mφ polarization in vivo. At E15, there was no significant difference between the two groups, possibly due to the continuous stimulation by apoptotic MEE cells (Fig. 9a, b). By E15.5, however, Mφs in the control palate were primarily M2, whereas those in the sonicated *P. gingivalis* group were predominantly M1 (Fig. 9a, c) corroborating our in vitro findings (Supplementary Fig. S7). We hypothesized that the different distributions of M1/M2 Mφs in the palate shelves of the two groups could also differentially influence surrounding MEPM cells through intercellular communication except MEE cells. Given that sEVs are the primary mediators of intercellular communication, we next examined the effects of M1 or M2 Mφs-derived sEVs on osteogenesis, the most distinct phenotype observed at E15.5.

### M2-sEVs promoted MEPM osteogenesis while M1-sEVs inhibited it in vitro

To investigate whether M1-sEVs or M2-sEVs play a role in MEPM osteogenesis, Raw 264.7 cells were polarized into M1 or M2 types by adding 40 ng/mL of IFN-γ and 20 ng/mL of IL-4, respectively. This significantly increased the expression of the M1 marker, CD86, or the M2 marker, CD206 (Fig. 9d). SEVs were then isolated from these M1 or M2 Mφs via ultracentrifugation and identified using transmission electron microscope (TEM), nanoparticle tracking analysis (NTA) and western blot analysis. As depicted in Supplementary Fig. S8a, the vesicles exhibited the typical cup-shaped morphology, with particle diameters ranging from 80 to 170 nm (Supplementary Fig. S8b). The inclusive markers of sEVs, TSG101 and CD63, were expressed, whereas the exclusive marker, Calnexin, was not expressed (Supplementary Fig. S8c).

To examine the impact of M1-sEVs and M2-sEVs on osteogenesis, MEPM cells were cultured in osteogenic induction medium containing either M1-sEVs or M2-sEVs. The protein levels of osteogenic markers, including bone morphogenetic protein 2 (BMP2), osteopontin (OPN), and Runx2, were measured 14 days post osteogenic induction. Among these markers, Runx2 expression was significantly inhibited by M1-sEVs but promoted by M2-sEVs in comparison to the control (Fig. 9e, f). ARS and quantitative calcium measurements displayed that mineralization was reduced in MEPM cells cultured with M1-sEVs compared to the control group at 21 days post osteogenic induction, 21 days following osteogenic induction. In contrast, cells cultured with M2-sEVs showed a significant increase in mineralization (Fig. 9g, h). These findings demonstrate that M2-sEVs enhance MEPM osteogenesis, while M1-sEVs inhibit it in vitro.

### Pharmacological ADAM17 inhibition rescued efferocytosis disorder, fusion failure, and osteogenic abnormities in the sonicated *P. gingivalis*-treated condition

**Fig. 10:**
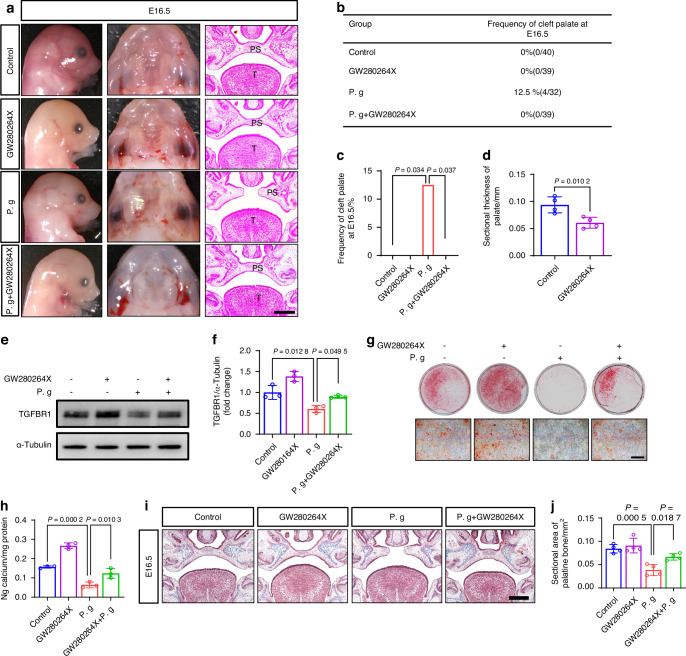
Pharmacological ADAM17 inhibition ameliorated osteogenic abnormities in the sonicated *P. gingivalis* (P. g)-treated condition. **a** Lateral and occlusal views and histological observation of mice palates after treated with P. g and (or) GW280264X. Bar, 200 μm, PS represents palatal shelves and T represents togue. **b** The frequency of cleft. **c** Frequency of cleft palate in P. g group compared with other groups, *P* value was calculated by Fisher’s exact test. **d** Quantification of the sectional thickness of embryonic palate in the control and GW280264X group. **e** Western blot analysis of TGFBR1, with (**f**) quantification of protein levels (*n* = 3). **g** Alizarin red S staining on 28 days and **h** the calcium concentration was determined by measuring the absorbance at 562 nm on a multiplate reader (*n* = 3). Bar, 500 μm. **i**, **j** Masson staining and statistical analysis (*n* = 4). Bar, 200 μm. The data are shown as the mean ± SD and were statistically analyzed by one-way ANOVA with Tukey’s multiple-comparison test (**f**, **h**, **j**) or two-tailed Student’s *t*-test (**d**). All the *P* values were two-sided and adjustments were made for multiple comparisons

**Fig. 11:**
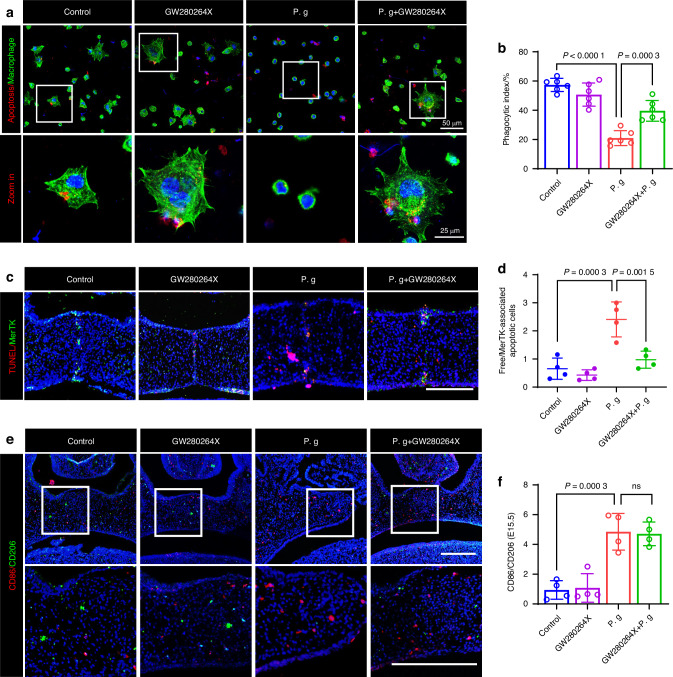
Pharmacological ADAM17 inhibition ameliorated efferocytosis disorder and fusion failure. **a** Representative fluorescent images showing engulfing of apoptotic MEE cells by macrophages and **b** phagocytic index based on the fluorescent image (*n* = 6). **c** Co-staining of MerTK positive macrophage (green) in TUNEL-positive regions (red), with **d** quantification of Free/MerTK-associated apoptotic cells (*n* = 4). Bar, 200 μm. **e** Co-staining of CD86 /CD206 and **f** quantification of CD86/CD206 at E15.5 (*n* = 4). Bar, 200 μm. The data are shown as the mean ± SD and were statistically analyzed by one-way ANOVA with Tukey’s multiple-comparison test (**b**, **d**, **f**). All the *P* values were two-sided and adjustments were made for multiple comparisons. P. g represents *P. gingivalis*

To evaluate whether the excessive expression of ADAM17 was causally related to the abnormal palatal fusion and osteogenesis induced by sonicated *P. gingivalis*, we administered GW280264X, an ADAM17 antagonist, to mice exposed to sonicated *P. gingivalis* from E10.5 to E14.5. In a limited sample size, the use of GW280264X reduced the incidence of CP from 12.5% to 0% under treatment with sonicated *P. gingivalis* (Fig. 10a–c, Supplementary Table 1, Supplementary Table 2). We also observed that the palatal morphology was relatively thin in the ADAM17 inhibitor group (Fig. 10a, d). In MEPM cells, the reduced expression of TGFBR1 was partially reversed (Fig. 10e, f). ARS and Masson staining further confirmed that the decreased osteogenesis in mice treated with sonicated *P. gingivalis* was significantly improved following ADAM17 inhibition, both in vitro (Fig. 10g, h) and in vivo (Fig. 10I, j). For Mφs, the diminished expression of MerTK (Supplementary Fig. S9a, b) and efferocytosis was partially restored both in vitro (Fig. 11a, b) and in vivo (Fig. 11c, d). However, the restorative effect of GW280264X on the average percentages of M1 Mφs and M2 Mφs per high-power field and their ratio in the palate were not significant (Fig. 11e, f, Supplementary Fig. S9c, d). Taken together, our findings indicate that the upregulation of ADAM17 due to glycolysis enhanced H4K12la, and the subsequent cleavage of TGFBR1 and MerTK, govern the CP with abnormal palate shelves fusion and osteogenesis caused by sonicated *P. gingivalis* during palate development.

## Discussion

Maternal periodontal disease has been reported to correlate with adverse pregnancy outcomes.^5,12^ A prior case-control study even revealed a surprisingly strong association between maternal periodontal disease and CP in offspring.^6^ However, the precise relationship has remained unclear for over a decade. Although a variety of periodontal pathogens are prevalent in the dental plaque of patients with periodontitis, it is noteworthy that the majority of periodontal pathogens identified in the placenta and amniotic fluid tissues of pregnant women experiencing adverse pregnancy outcomes, such as eclampsia and premature delivery, are predominantly *P. gingivalis*.^12^ Consequently, we selected *P. gingivalis* as the focal point of our research.

In our study, we exposed pregnant mice to sonicated *P. gingivalis* via both intravenous injection and oral administration starting from E2.5, as periodontal disease is characterized by both endotoxemia and the ingestion of periodontal bacteria.^24,33^ We found that treatment with sonicated *P. gingivalis* resulted in CP with cleft between the palate shelves and reduced osteogenesis. At E15, the incidence of residual epithelial remnants at the midline without any mesenchymal confluency in the sonicated *P. gingivalis* group was 21.4%. However, CP was seen in 12.1% of the litters when evaluated at E16.5.

We posit two possible explanations for this frequency disparity. Firstly, given that previous studies reported an association between *P. gingivalis* infection and spontaneous abortion, we hypothesize that severely affected fetal mice at E15 might have ultimately undergone abortion or embryonic absorption by E16.5 as it might influence not only palate development, but also the whole body.^12^ During the course of the experiment, it was additionally observed that embryos exposed to *P. gingivalis* exhibited various craniofacial, ocular and dermatological abnormalities. Secondly, the mating time might vary between different female mice by several hours, and offspring within the same pregnant mice could even develop at different rates. Consequently, at E15, some late-mating or slower-developing offspring might not have initiated epithelial apoptosis at the midline, but they were still included in the abnormal phenotype count. To eliminate the influence of variable developmental periods, we selected offspring with no signs of developmental delay for our subsequent study based on dental germ morphology.

To investigate the mechanisms of CP underlying the abnormal palate shelves fusion and osteogenesis caused by sonicated *P. gingivalis*, we further examined the impact of sonicated *P. gingivalis* on MEPM cells and Mφs, respectively. A crucial aspect of cellular physiology that has recently garnered attention in embryonic development is the regulation of glucose metabolism. We discovered that both MEPM cells and Mφs underwent a metabolic shift from OXPHOS to glycolysis to cope with *P. gingivalis* exposure. This shift is contrary to the typical metabolic changes seen during palate development, leading to metabolic disorder.^11^ Considering previous studies that have identified a link between glycolysis and inflammation,^34,35^ we hypothesize that metabolic alterations may be instigated by inflammation (see Supplementary Fig. S10). This hypothesis warrants further exploration.

Most metabolic changes can indirectly determine cell identities by epigenetic regulation.^8,19^ More recently, glycolysis-derived lactate was identified as a substrate for histone lactylation and lactate-derived histone lactylation was shown to stimulate gene expressions.^20^ Zhang et al. discovered that *P. gingivalis* msRNAP.G_45033 could induce Aβ production by enhancing glycolysis and histone lactylation in Mφs.^36^ Consist with this finding, in this research, we demonstrated that an increase in the lactate pool due to sonicated *P. gingivalis* facilitates glycolysis, then enhanced histone lactylation which played a significant role in defining ADAM17 binding, as indicated by H4K12la and ADAM17 ChIP-qPCR analysis of MEPM cells and Mφs.

Previous research has indicated that H4K12la specifically upregulates multiple genes related to cell proliferation.^37^ Consistent with these findings, ADAM17 has been reported to play a crucial role in cell proliferation during development. Earlier studies on the developing cerebellum and spinal cord found that the expression level of ADAM17 was higher during early stages of development—when cell proliferation is predominant—and gradually declines in response to environmental and functional changes.^38–40^

In our study, we observed that the palatal morphology was relatively thin in the ADAM17 inhibitor group, suggesting that ADAM17 might also play a significant role in cell proliferation during palate development. Furthermore, we noted that the expression of ADAM17 gradually decreased with maturation during normal palate development. However, in the sonicated *P. gingivalis*-treated group, H4K12la facilitated the transcription of ADAM17, disrupting its usual temporal expression pattern (Supplementary Fig. S5). The sustained high expression of ADAM17 consequently mediated the shedding of MerTK in Mφs and TGFBR1 in MEPM cells, leading to abnormal efferocytosis and osteogenesis in palate development. Our findings, along with previous studies, suggest that glucose metabolism and its derivative histone lactylation fine-tune the temporal expression of ADAM17, underlining the pivotal role of metabolism-associated epitranscriptomic machinery in coordinating organ growth and functional maturation timing during palate development.

Palate shelves fusion and wound repair are regenerative processes sharing common signaling pathways and gene regulatory networks.^41^ Previous studies demonstrated Mφ-mediated efferocytosis played a prominent role in wound healing by removing apoptotic cells and then dampening inflammation and secreting factors that regulate the proliferation.^42^ The normal exercise of efferocytosis can induce the Mφ reprogramming, so that Mφ transition from pro-inflammatory M1 to reparative M2 for tissue repair.^32^ To determine whether Mφs play a similar role in palatal fusion, Christiaan et al. investigated whether palatal fusion was disrupted in heme oxygenase-2 knockout (HO-2 KO) mice due to altered epithelial cell and Mφ interactions within the MES and they found HO-2 KO Mφs were still functional in phagocytosing apoptotic fragments, with no disturbance was observed in palatal fusion in HO-2 KO foetuses. This may be due to their examination of Mφ function in a non-pathological environment, with the heightened expression of HO-1 possibly compensating for the deletion of HO-2 in Mφs recruited to the fusing palate shelves.^16^

In contrast, our study provides additional evidence that under pathological conditions, disrupted efferocytosis due to MerTK shedding hinders the clearance of apoptotic epithelium in the MES, resulting in a cleft between the palate shelves. These observations emphasize the significant influence of the pathological environment on Mφ function, a situation analogous to inflammatory stresses in the wound healing context. Given that palatogenesis and wound healing share many key cellular behaviors, deepening our understanding of one can inform that of the other, leading to the development of novel therapeutic strategies.

Moreover, we observed that the weakened efferocytosis inhibited the phenotypic transformation of Mφs, led to a predominant distribution of M1 Mφs at E15.5, which might further impact MEPM osteogenesis through sEV-mediated cross-talk. Interestingly, among the detected osteogenic markers, the expression of Runx2 but not BMP2 and OPN was significantly regulated by M1-sEVs or M2-sEVs. Since previous studies demonstrated BMPs functioned by activating SMAD proteins to stimulate expression of Runx2 which induced expression of osteoblast markers such as collagen type I, ALP and OCN,^43,44^ we speculated that M1-sEVs or M2-sEVs might act downstream of BMP2, while the insignificant variation of OPN might be due to its not being the main target gene for direct binding of Runx2 in MEPM cells. Therefore, understanding the sEV-mediated interaction among different type of cells especially investigating the Runx2 function in more detail will facilitate the cognition of palatogenesis and development of targeted drugs for CP in the future.

Additionally, given our findings that pharmacological inhibition of ADAM17 using GW280264X ameliorated the aberrant efferocytosis induced by *P. gingivalis*, we subsequently investigated whether such inhibition could also rescue the abnormal polarization of Mφs and enhance osteogenic differentiation. However, the restorative effect of GW280264X on the Mφ phenotypic shift was not significant, possibly due to the challenge of phenotypic switching in the chronic inflammatory environment induced by sonicated *P. gingivalis*. These results suggested that future research should undertake a more detailed investigation at the molecular level to identify specific substances within *P. gingivalis*, as well as components in other pathogens, that may activate the embryonic inflammatory environment and induce Mφ phenotype transformation.

Although Mφ polarization remained unchanged, GW280264X successfully ameliorated *P. gingivalis*-induced osteogenesis abnormalities. This finding confirmed that the elevated expression of ADAM17 induced by *P. gingivalis* could also influence osteogenesis through alternative pathways. Supporting evidence for this assertion is provided by our findings, which illustrate that, beyond the indirect osteogenic regulation facilitated by sEVs, direct regulation induced by sonicated *P. gingivalis* also results in aberrant osteogenesis. This phenomenon is attributed to the shedding of TGFBR1 in MEPM cells.

TGFBR1 plays important roles in morphogenesis of many craniofacial tissues. Dudas et al. reported missing and dysplastic craniofacial skeletal structures in *Tgfbr1/Wnt1*-Cre mutants, which deleted Tgfbr1 only in cells that express *Wnt1*-Cre, i.e., in neural crest cells, and in the neural plate. They suggested that the observed phenotypes in *Tgfbr1/Wnt1*-Cre mutants could be caused by defective cell survival.^27^ However, in our study, we did not observe the negative regulation of sonicated *P. gingivalis* on cell survival. On the contrary, both in vitro and in vivo results showed that sonicated *P. gingivalis* promoted the proliferation of MEPM cells and inhibited the apoptosis. Since early studies have shown that bacteria-induced metabolic reprogramming to glycolysis promotes cell proliferation as a strategy for bacterial initiation of disseminated infection,^45,46^ and given the association between ADAM17 and cell proliferation,^38–40^ we suggested that the effects of metabolic reprogramming and subsequent ADAM17 upregulation might counteract the negative regulation of TGFBR1 on cell survival. We found that although the density of MEPM cells was not reduced, the shedding of TGFBR1 led to an obstruction of MEPM cells migration and osteogenic differentiation, ultimately leading to osteogenic abnormalities.

**Fig. 12:**
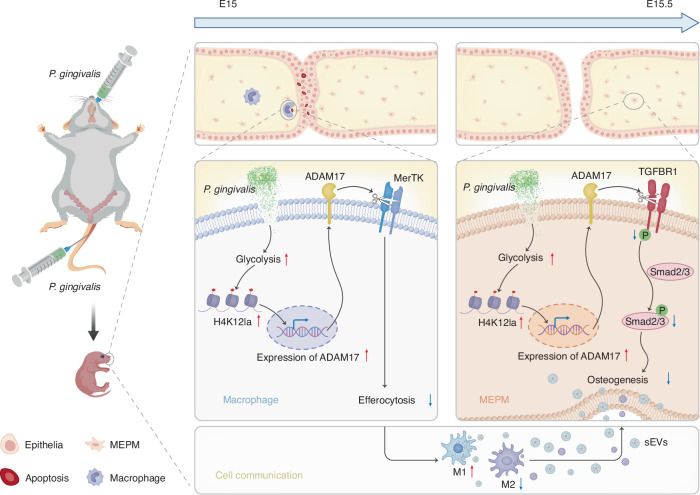
Schematic diagram of the etiology of CP caused by sonicated *P. gingivalis*. Glycolysis and H4K12la were enhanced in both macrophages and MEPM cells under *P. gingivalis* exposure which further promoted the transcription of ADAM17, subsequently mediated the shedding of MerTK in macrophages and TGFBR1 in MEPM cells and resulted in the suppression of efferocytosis and osteogenesis in fetal palate, eventually caused abnormalities in palate fusion and ossification. The abnormal efferocytosis also led to a predominance of M1 macrophages, which indirectly inhibited palatal osteogenesis via sEVs

In conclusion, we demonstrate the etiology of CP caused by sonicated *P. gingivalis* by upregulation of ADAM17 due to glycolysis enhanced H4K12la, and the resulting cleavage of TGFBR1 and MerTK, underlie the abnormal palatal fusion and osteogenesis during palate development (Fig. 12). This may explain the strong association between maternal periodontal disease and CP in their children. Since the molecular processes and signaling pathways associated with palatal ontogeny are mirrored in the embryogenesis of multiple other systems,^17,47^ understanding the pathological mechanisms underlying palate development during maternal periodontal disease will also allow us to explore potential targets for preventing or treating infection-induced congenital birth defects.

## Materials and methods

### *P. gingivalis* culture

*P. gingivalis* (ATCC 33277) was cultured following a previously described method.^48^ A bacterial suspension of 10^9^ CFU/mL was sonicated at 300 W for 90 cycles (10 s on, 10 s off) on ice using an ultrasonic cell disruptor (JYD-150, Zhixin Instrument Co., China), ensuring thorough bacterial lysis. The sonicated extract was then sterilized by filtration through 0.22-μm-pore-size filter. The total protein concentration was measured, and the solution was stored at −80 °C.

### Cell culture

MEPM cells were obtained from E15.5 ICR pregnant mice as previously described.^11^ The cells were cultured in the Dulbecco’s Modified Eagle Medium/F12 (DMEM/F12; SV30023.01, Hyclone, USA) containing 10% fetal bovine serum (FBS; 10099-141, Gibco, USA) and 1% penicillin/streptomycin (C100C5, NCM Biotech, China).

The RAW264.7 murine macrophage cell line (CL-0190, Procell, China) was purchased from Procell company and cultured in Raw264.7 specialized medium (CM-0190, Procell, China).

BMDM were prepared as previously described^49^ and cultured for 7 d in RPMI 1640 medium (SH30809.01, HyClone, USA) with 10% FBS and 1% penicillin/streptomycin and 10 ng/mL M-CSF (81627-83-0, Sigma-Aldrich, USA).

The MEE cell line (BFN607200453, DSMZ Cell Bank, China) were obtained from the cell bank of Chinese Academy of Sciences, and cultured in DMEM medium (SH30022.01, HyClone, USA), supplemented with 15% FBS and 1% penicillin–streptomycin.

### Preparation and characterization of sEVs

M1-sEVs and M2-sEVs were extracted and isolated and characterized as described previously.^50^ The morphology of sEVs was identified using TEM. The concentration and particle size distribution of sEVs were analyzed by NTA, and the surface sEV-markers were tested by western blot. 100 μg/mL sEVs were used to treat MEPM cells.

### Mice experiments

Healthy female C57BL/6 mice (8–10 weeks old; weight, 22–25 g) were paired with healthy males (12–14 weeks old) in a 2:1 ratio overnight. The presence of vaginal plugs was checked the next morning and marked as day 0.5 of gestation (E0.5). The mice were randomly divided into two groups: those administered sonicated *P. gingivalis* suspension in saline and those receiving saline only. Experimental periodontal disease was induced as previously described.^24^ Administration was carried out through both oral gavage and intravenous injection in each group. A sonicated *P. gingivalis* suspension (10^8^ CFU in 100 µL saline) and saline only (100 µL) were administered by the two methods, respectively. Administration was performed every other day from E2.5. Pregnant mice were dissected at E13.5, E14.5, E15, E15.5, and E16.5 and euthanized by cervical dislocation (*n* = 6 at each time point per group), and fetal litters were removed via dissection to observe palate development. The embryo samples were embedded for histological sections. For GW280264X (HY-115670, MCE, USA) treatment, pregnant mice were injected with the working solution (0.1 μg/g body weight) from E10.5 to E14.5.

### ELISA assays for RgpA

Amniotic fluid and fetal palate tissue were collected from four pregnant mice at each designated time point. These samples were subsequently analyzed and quantified for the presence of *P. gingivalis* gingipain R1 (RgpA) using an RgpA-specific antibody (orb243611, biorbyt, UK) and Ancillary Reagent Kit (E-ELIR-K001, Elabscience, China) following the manufacturer’s protocol.

### Histological analysis

Palate tissue histological sections were sliced to a thickness of 5 µm using a rotary microtome, from the nasal cavity to the ear. The sections were stained with HE and Masson following methods described previously.^11^ Microphotographs were captured using a digital camera attached to a microscope (BX61, Olympus, Japan). The collagen volume was measured using ImageJ.

### Immunohistochemistry

All sections underwent antigen activation. Post washing and blocking, the slides were incubated overnight at 4 °C with the primary antibody (TGFBR1, ab288303, Abcam, UK; PCNA, 10205-2-AP, Proteintech, China), diluted 1:100 in PBS. The sections were rinse, then incubated with secondary antibodies (goat anti-rabbit IgG-HRP; PV9001, ZSGB-BIO, China) and diluted 1:400 in PBS for 1 h. Following another round of washing, the sections were detected using a standard DAB detection system (ZLI-9019, ZSGB-BIO, China). The quantification of positive cells was measured using ImageJ.

### Assessment of apoptosis and efferocytosis in the medial edge seam

Apoptotic cells in the medial edge seam were examined using an in situ TUNEL kit. MerTK, a member of the TAM family (Tyro3, Axl, and MerTK), is known for its contribution to the regulation of Mφ efferocytosis. Mφ efferocytosis in this study was assessed by co-staining of apoptotic cells and MerTK+ Mφs, following a previous protocol. Mφs were stained with a rabbit anti-MerTK antibody (ab52968, Abcam, UK, final dilution, 1:50) and then a FITC-labeled goat anti-rabbit IgG (35552, Thermo Fisher, USA, final dilution, 1:200). The ratio of free to MerTK-associated apoptotic cells was calculated. Images were captured using identical exposure settings and quantified using ImageJ.

### Fluorescence multiplex immunohistochemical analysis

Fluorescence multiplex immunohistochemical analysis was performed according to the manufacturer’s instructions. The sections underwent three rounds of staining in the order of H4K12la (PTM-1411, PTM BIO, China, 1:150), ADAM17 (ab39162, Abcam, UK, 1:150), MerTK (ab52968, Abcam, UK, 1:150), or TGFBR1 (ab288303, Abcam, UK, 1:150) each employing a separate fluorescent tyramide signal amplification system (Quadruple-Fluorescence Immunohistochemical Mouse/Rabbit Kit [RS0037]; ImmunoWay, USA). Images were captured by confocal laser scanning microscopy (TSC SP5, Leica, Germany) using identical exposure settings and quantified using ImageJ.

### In vitro efferocytosis analysis

MEE cells were resuspended in DMEM medium supplemented with 15% FBS and 0.5 μmol/L actinomycin D and incubated for 12 h. At this time, 85% of the MEPM cells were apoptotic. Apoptotic MEE cells were stained with DiI fluorescent dye (D8700, Solarbio, China), following the manufacturer’s instructions. Raw 264.7 cells (1 × 10^5^ cells per well) were plated in 12-well plates. After a 24-h incubation, the cells were exposed to DiI-labeled apoptotic for 1 h. The ratio of Raw 264.7 cells to MEE cells was 10:1. Undigested cells were removed by washing three times with ice-cold PBS, and Raw 264.7 cells were stained with Phalloidin. Efferocytosis of apoptotic cells was visualized using fluorescence microscopy.

### CCK-8 assay

Third-generation palate cells were plated into 96-well plates at a density of 7 × 10^3^ cells per well with six independent replicates. After the cells adhered to the wall, 10 μL of CCK-8 reagent (CK04; Dojindo Laboratories) was added separately to each well at 0, 24, 48, and 72 h, followed by incubation at 37 °C for 2 h. Each sample was analyzed at 450 nm with a SpectraMax Paradigm microplate reader (10822-512; Molecular Devices, USA).

### Apoptosis assay

About 2.5 × 10^5^ cells per well were seeded in 6-well plates and incubated at 37 °C. The following day, cells were harvested using trypsin digestion without EDTA, and then stained with annexin V-fluorescein isothiocyanate (FITC) and PI following the instructions of the FITC-Annexin V Apoptosis Detection Kit (C1062L; Beyotime, China). Cell analysis was carried out via flow cytometry (Accuri C6; BD, USA).

### Scratch test

About 3 × 10^5^ cells per well were seeded into 6-well plates. A 1-ml pipette tip was used to horizontally scratch the plate on the next day. Images were obtained using an inverted phase-contrast microscope after 24 and 48 h. The migration areas were detected and calculated using Image-Pro Plus 6.0

### Western blot

Cells were washed thrice with ice-cold PBS and lysed in RIPA (C1053, Applygen, China) buffer containing protease inhibitor cocktail (PIC; P8340, Sigma-Aldrich, USA) and phenylmethylsulfonyl fluoride (PMSF; 93482, Sigma-Aldrich, USA) for 10 min on ice before being centrifuged at 14 000 × *g* for 15 min at 4 °C. Protein samples were resolved on 10% SDS–PAGE gels and subsequently transferred onto polyvinylidene difluoride membranes. Membranes were blocked in 5% (w/v) bovine serum albumin (BSA) in Tris-buffered saline-Tween for 1 h at room temperature, and then incubated with primary antibodies (listed in Supplementary Table 3, final dilution 1:1 000) followed by the appropriate secondary antibody. Proteins were detected by enhanced chemiluminescence detection reagents (P10300, NCM 377 Biotech, China). The quantification of protein level was measured using ImageJ.

### Quantitative real-time polymerase chain reaction (qRT-PCR)

Total RNA was extracted using TRIzol (CW0580; ComWin Biotech, China) and converted to cDNA using the HiScript II 1st Strand cDNA Synthesis Kit (Vazyme, R233-01, China). Expressions was analyzed in triplicate using qRT-PCR instrument (CFX96 Touch Real-Time PCR Detection System; Bio-Rad Laboratories, USA) and MagicSYBR Mixture (CW2601H; ComWin Biotech, China). The final results were normalized to β-actin levels and analyzed by calculating the comparative cycle threshold values (2^−ΔΔCt^). All primer sequences are listed in Supplementary Table 4.

### Chromatin immunoprecipitation followed by quantitative PCR (ChIP-qPCR)

ChIP-qPCR was measured using the ChIP assay Kit (P2078, Beyotime, China) and performed according to the manufacturer’s instructions. For immunoprecipitation, the diluted chromatin was incubated with H4K12la antibodies (listed in Supplementary Table 3, final dilution 1:50) overnight at 4 °C with continuous rotation, followed by a 2-h incubation with 50 ml of 50% (v/v) protein A/G and washed with TSE I, TSE II, and TSE III buffers. The pulled-down chromatin complex was eluted by TE, and both eluted and input samples were de-crosslinked at 55 °C for 12 h in elution buffer. DNA was purified with the QIAquick PCR Purification Kit. qPCR was performed as previously described, and the primer sequences for ChIP-qPCR are listed in Supplementary Table 4.

### Alkaline phosphatase (ALP) and Alizarin red staining (ARS)

ALP staining was measured using the BCIP/NBT Alkaline Phosphatase Color Development Kit according to the instruction from manufacturer’s protocol (C3206, Beyotime, China). ALP activity assay was performed using an ALP activity kit according to the instruction from manufacturer’s protocol (245-325-0, Sigma-Aldrich, USA). Signal strength was normalized based on protein concentration. For ARS, MEPM cells were fixed for 30 min and then stained with 1% alizarin red (A5533, Sigma-Aldrich, USA) at room temperature. For quantification, calcium mineralization was measured as described in prior literature.^51^

### Statistical analyses

Statistical analyses were performed using Prism 8 (GraphPad Software) and data are presented as mean ± SD. An unpaired two-tailed Student’s t-test was used to determine significance between two groups of normally distributed data. For comparisons between multiple groups, an ordinary one-way or two-way ANOVA was used, followed by Tukey’s test. Comparisons of CP frequency were performed using Fisher’s exact test. The statistical tests used for each experiment are indicated in the figure legends. A value of *P* < 0.05 was considered statistically significant. All in vitro experiments were performed in triplicate. Animal feeding, treatments and histological analyses were performed in a single-blinded fashion.

### Ethics approval and consent to participate

All animal experimentation was approved by the Animal Care and Use Committee at Beijing Stomatological Hospital, affiliated with Capital Medical University (permit number: KQYY-202208-003, Beijing, China).

## Supplementary information

Supplementary Information

## Data Availability

The data supporting the findings of this study are available from the corresponding author upon reasonable request.
