# Supplementary material for: Administration of Porphyromonas gingivalis in pregnant mice enhances glycolysis and histone lactylation/ADAM17 leading to cleft palate in offspring
Source: Int J Oral Sci. 2025 Mar 13;17:18. doi: 10.1038/s41368-025-00347-x (PMC11903673; doi:10.1038/s41368-025-00347-x)
Supplement: Supplementary file 1 — Supplementary Information [file 41368_2025_347_MOESM1_ESM.docx]

**Supplementary Information**

**Administration of** ***Porphyromonas Gingivalis* in Pregnant Mice Enhances Glycolysis and Histone Lactylation/ADAM17 Leading to Cleft Palate in Offspring**

Xige Zhao^1^, Xiaoyu Zheng^1^, Yijia Wang^1^, Jing Chen^1^, Xiaotong Wang^1^, Xia Peng^1^, Dong Yuan^2^, Ying Liu^1^, Zhiwei Wang^1^, Juan Du^1，2*^

1 Laboratory of Orofacial Development, Laboratory of Molecular Signaling and Stem Cells Therapy, Molecular Laboratory for Gene Therapy and Tooth Regeneration, Beijing Key Laboratory of Tooth Re-generation and Function Reconstruction, Capital Medical University School of Stomatology, Tiantan Xili No.4, Beijing 100050, China

2 Department of geriatric dentistry, Capital Medical University School of Stomatology, Tiantan Xili No.4, Beijing 100050, China

* Correspondence: Juan Du, email: juandug@ccmu.edu.cn; Tel.: 86-10-57099319

**Supplemental Figures**

**
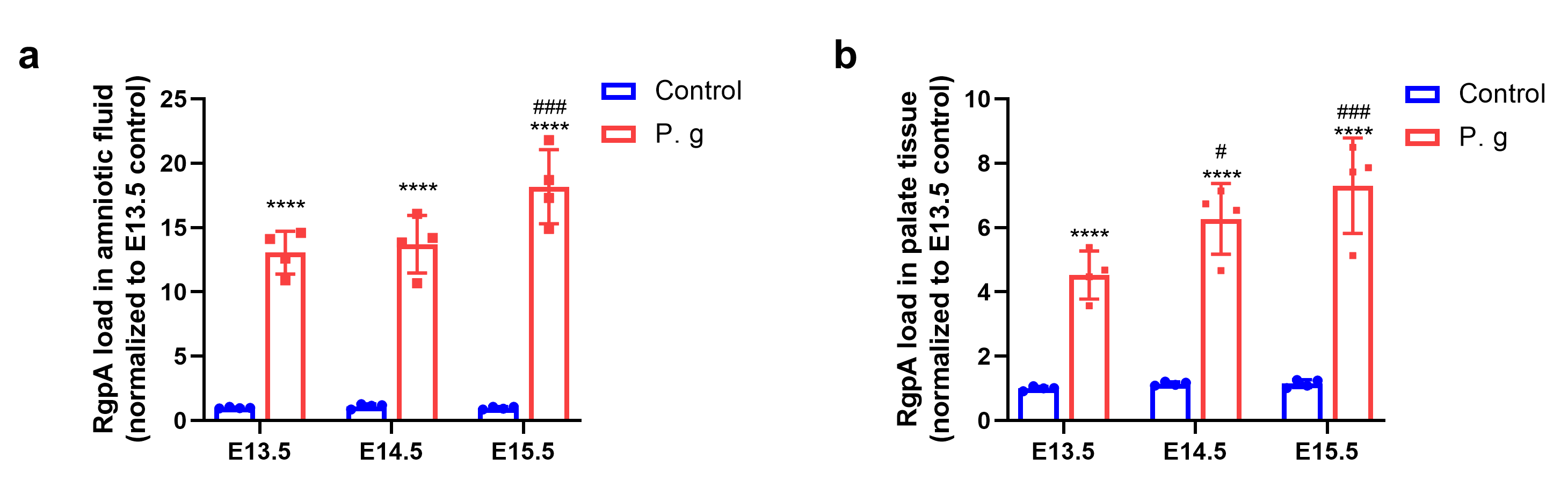
**

**Supplemental Figure S1.** ELISA monitoring the level of *P. gingivalis* gingipain R1 (RgpA) load in amniotic fluid **(a)** and palate tissue **(b)** from E13.5 to E15.5 (n=4). ^#^ represents a comparison with E13.5 of the same treated method, * represents a comparison between the two groups within a time point. ^###^*P* < 0.001, ****^/####^*P* < 0.0001. The data are shown as the mean ±SD and were statistically analysed by one-way ANOVA with Tukey’s multiple-comparison test. All the *P* values were two-sided and adjustments were made for multiple comparisons.


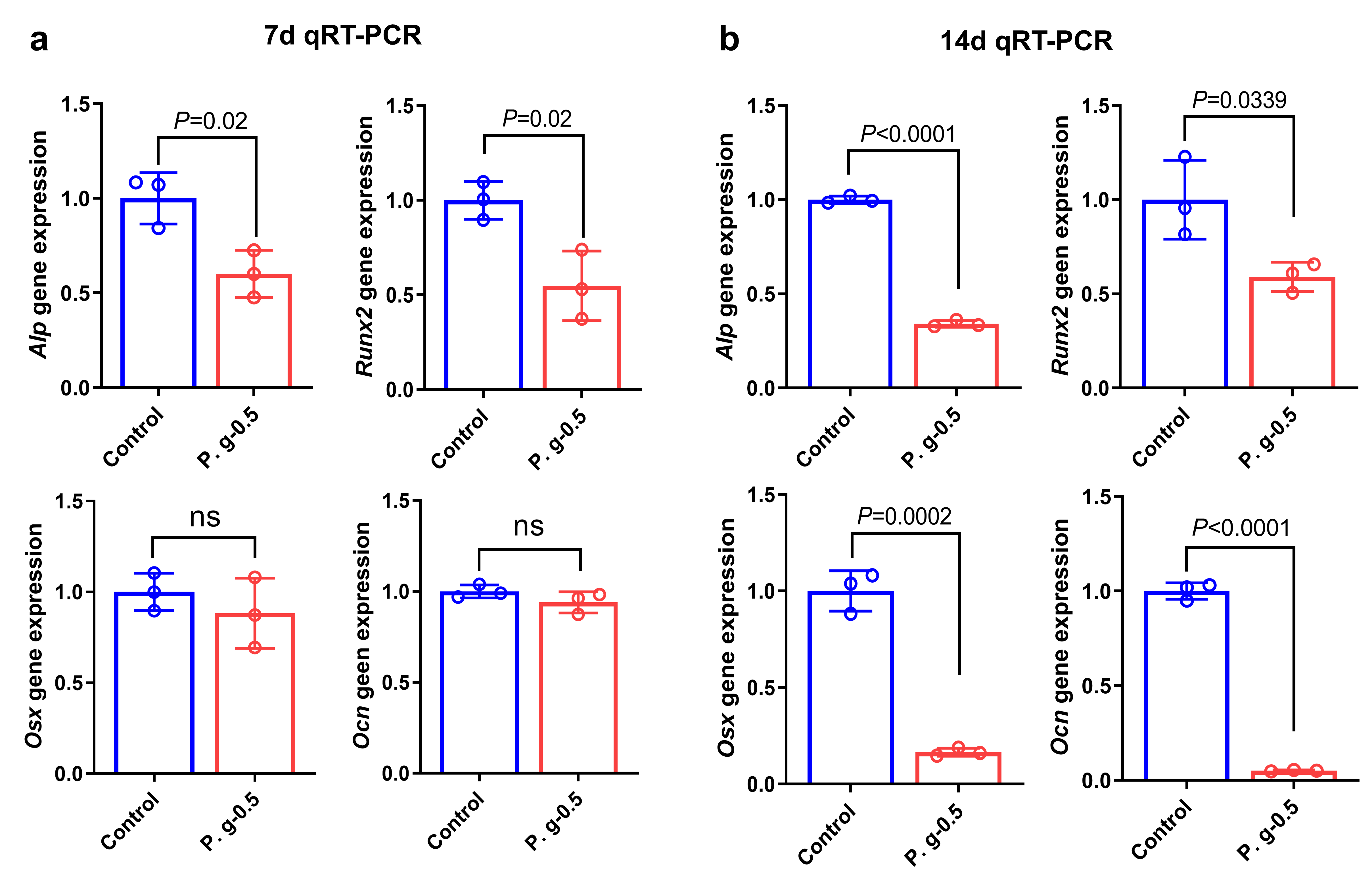


**Supplemental Figure S2.** qPCR assays monitoring expression of the osteogenic genes in MEPM cells after osteogenic induction for 7 days **(a)** and 14 days **(b)** (n=3). The data are shown as the mean ±SD and were statistically analysed by two-tailed Student’s t-test.


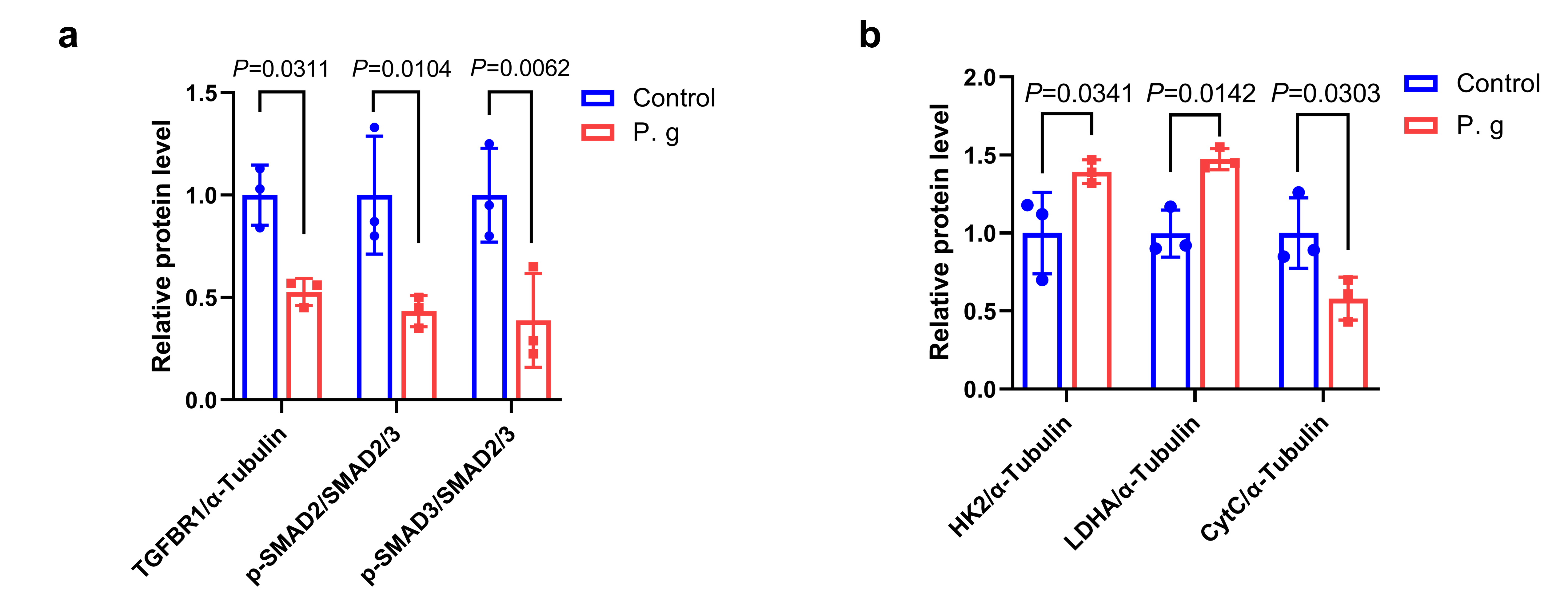


**Supplemental Figure S3. (a)** Quantification of the protein in classical TGFβ pathway in MEPM cells (n=3). **(b)** Quantification of the markers in glycolysis and OXPHOs in MEPM cells (n=3). The data are shown as the mean ±SD and were statistically analysed by one-way ANOVA with Tukey’s multiple-comparison test. All the *P* values were two-sided and adjustments were made for multiple comparisons.


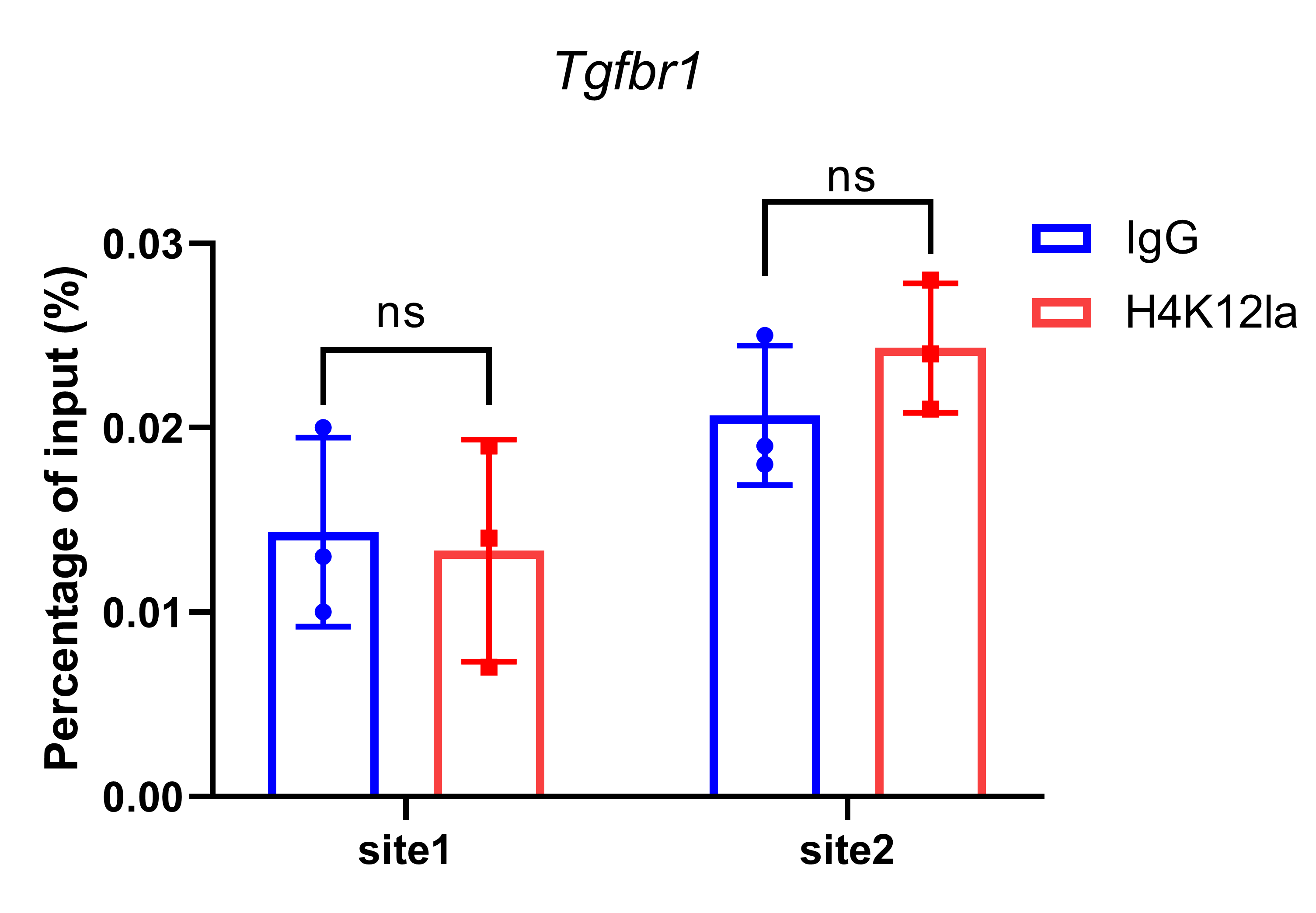


**Supplemental Figure S4.** ChIP-qPCR analysis of the *Tgfbr1* promoters was performed using antibodies against H4K12la in MEPM cells (n=3). The data are shown as the mean ±SD and were statistically analysed by two-tailed Student’s t-test.


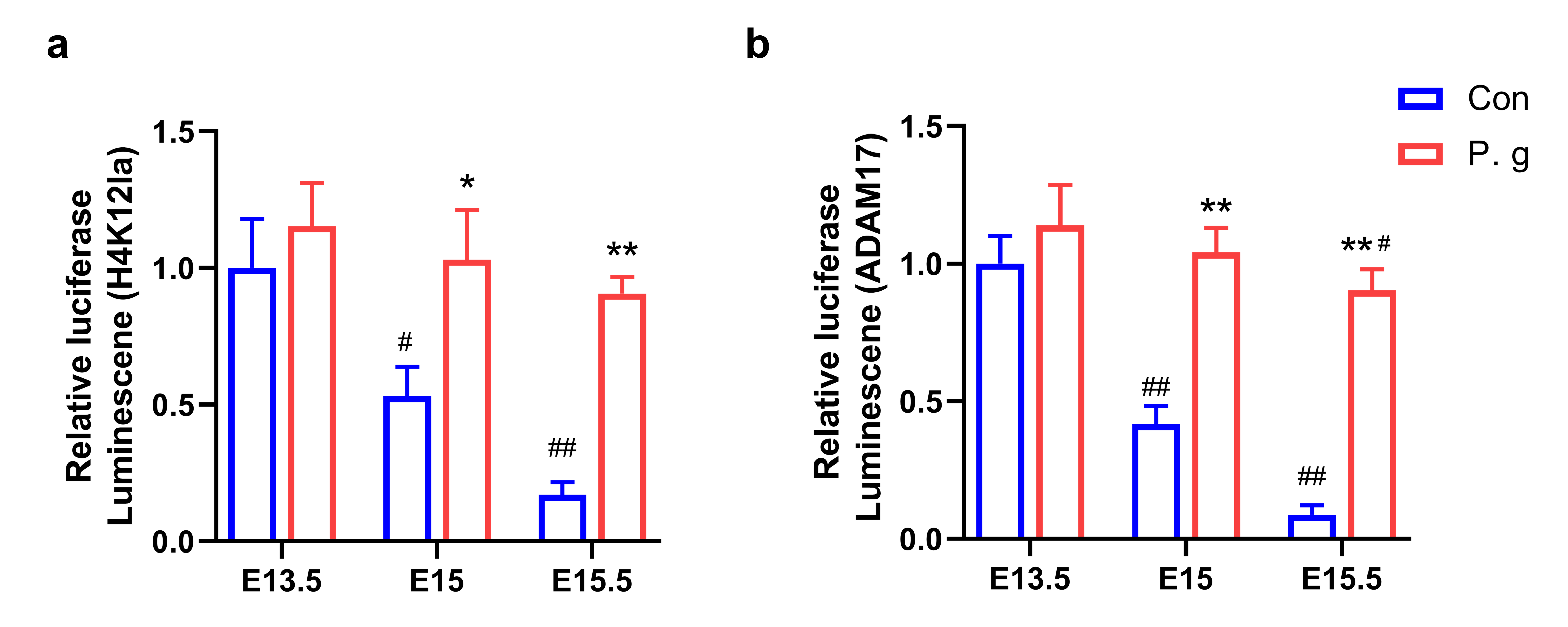


**Supplemental Figure S5.** Quantification of H4K12la **(a)** and ADAM17 **(b)** intensity based on Fig. 4a. ^#^ represents a comparison with E13.5 of the same treated method, * represents a comparison between the two groups within a time point. *^/#^*P* < 0.05, **^/##^*P* < 0.01. The data are shown as the mean ±SD and were statistically analysed by one-way ANOVA with Tukey’s multiple-comparison test. All the *P* values were two-sided and adjustments were made for multiple comparisons.


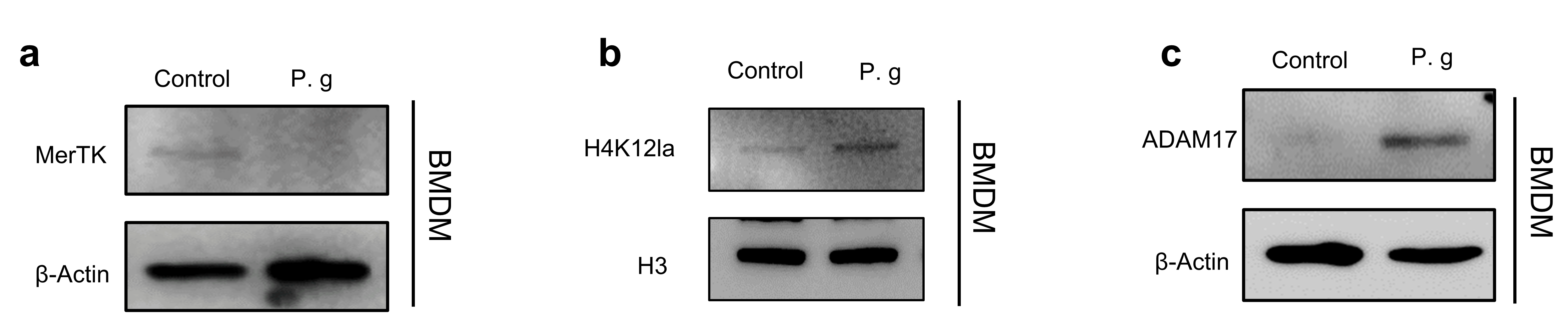


**Supplemental Figure S6.** Western blot assays monitoring expression of the H4K12la/ADAM17/MerTK in mice bone marrow derived-macrophages treated with or without sonicated *P. gingivalis*.


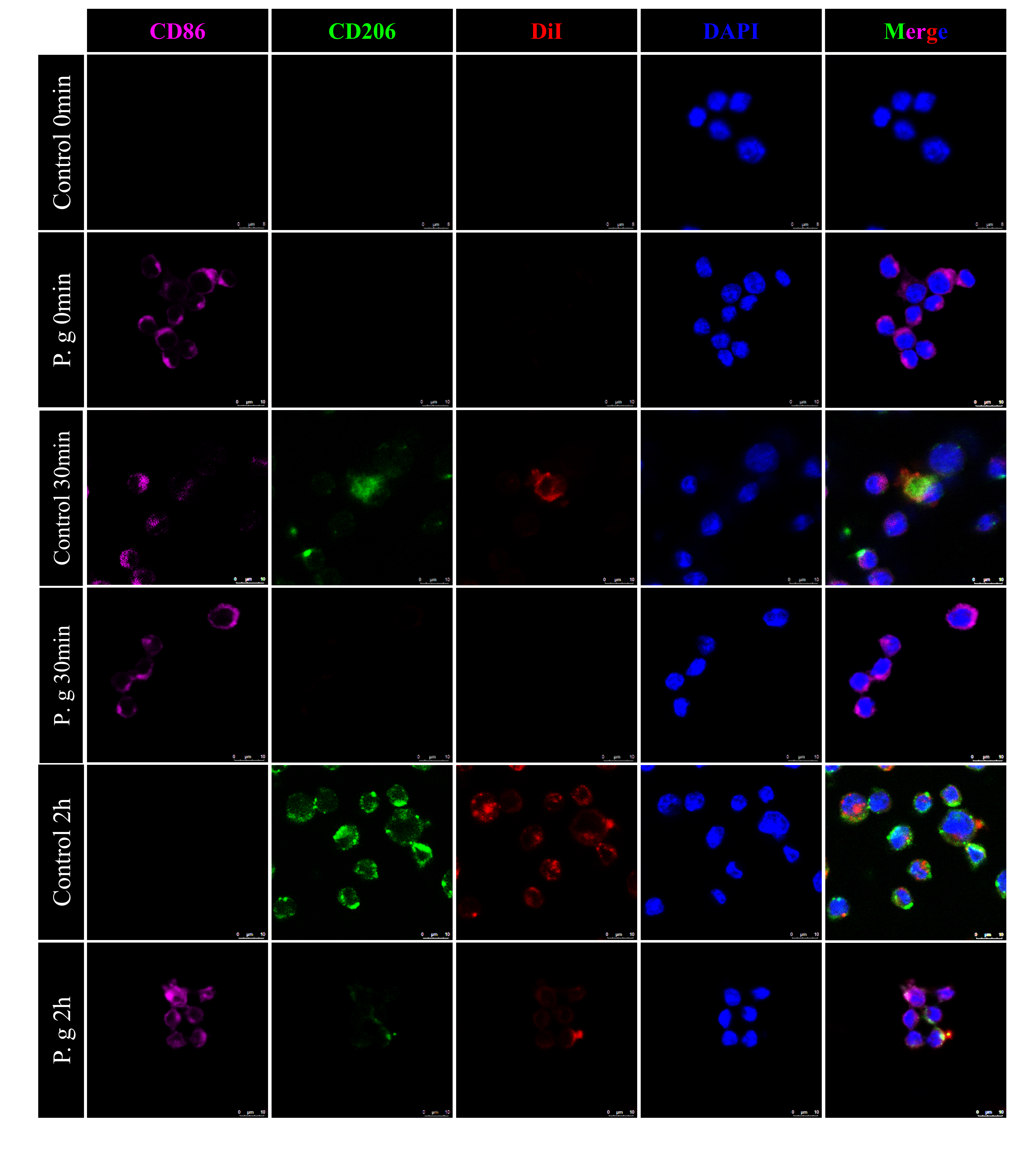


**Supplemental Figure S7.** *In vitro* co-staining of CD86/CD206 in “macrophage and apoptotic MEE co-culture system” showed Raw 264.7 macrophages pretreated with sonicated *P. gingivalis* cannot undergo a phenotypic switch upon efferocytosis.


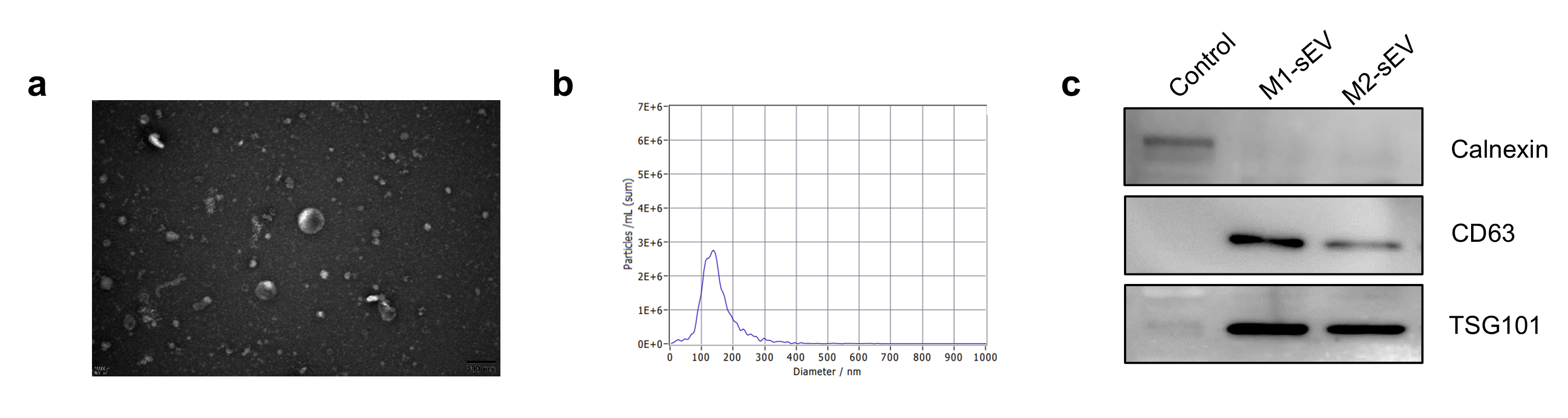


**Supplemental Figure S8.** SEVs from M1 or M2 macrophages were identified using **(a)** TEM, **(b)** NTA and **(c)** western blot analysis.


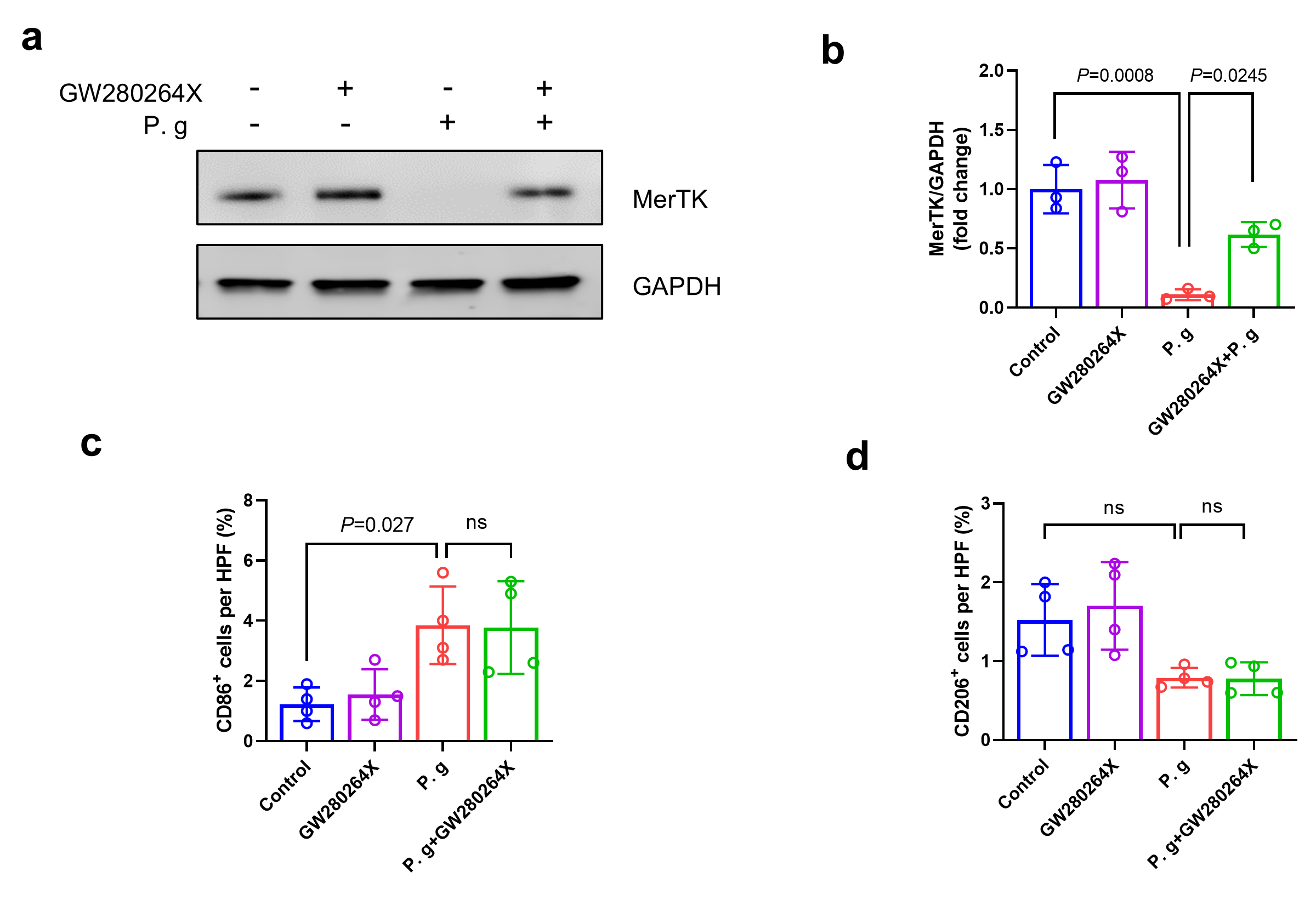


**Supplemental Figure S9.** **(a)** Western blot analysis of MerTK after treated with sonicated *P. gingivalis*. and (or) GW280264X, with **(b)** quantification of protein levels (n = 3). (**b)** Quantification of the average percentages of CD86^+^ cells (M1 Mφs) per HPF (n = 4). **(c)** Quantification of the average percentages of CD206^+^ cells (M2 Mφs) per HPF (n = 4). The data are shown as the mean ±SD and were statistically analysed by one-way ANOVA with Tukey’s multiple-comparison test (b, c, d). All the *P* values were two-sided and adjustments were made for multiple comparisons.


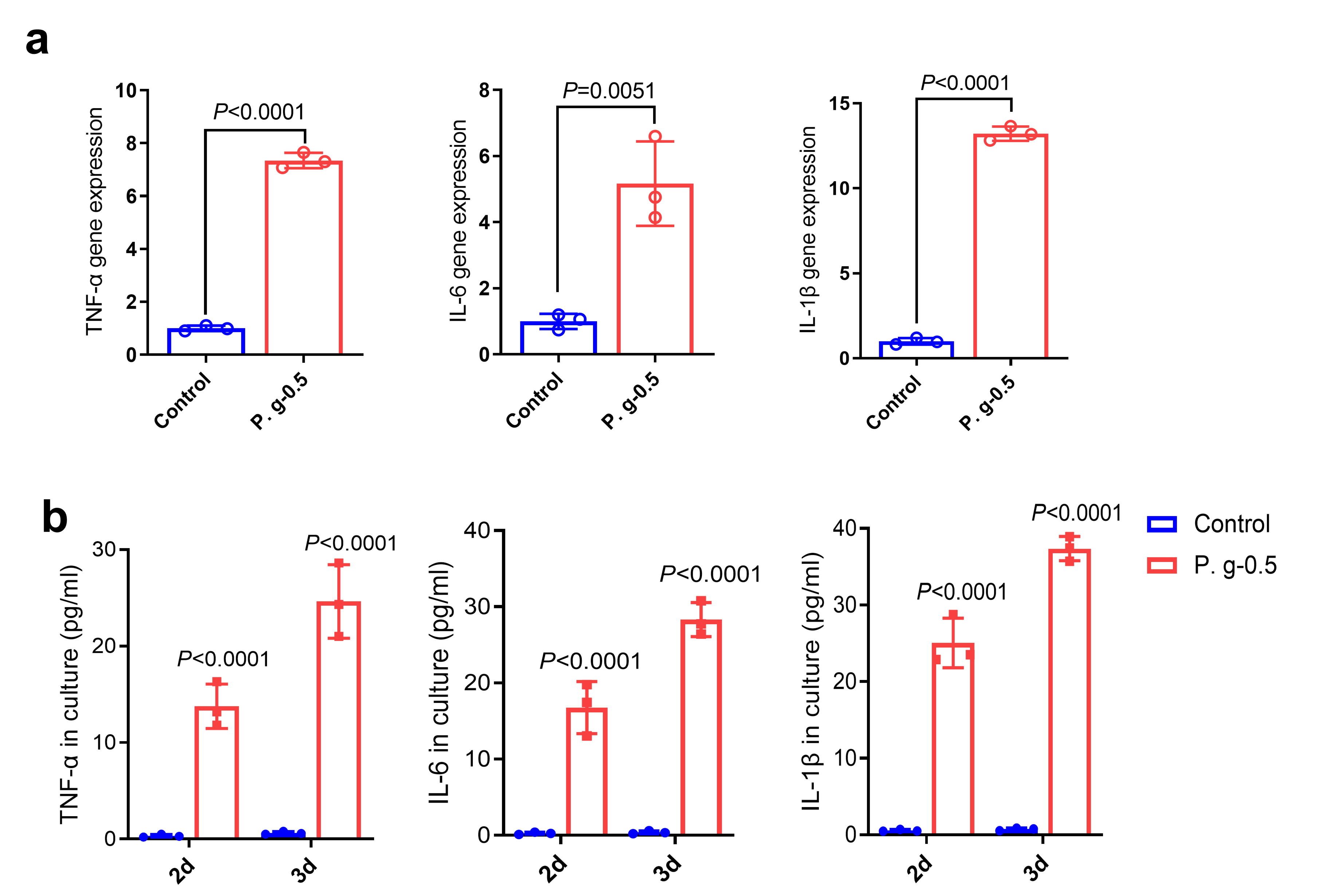


**Supplemental Figure S10.** Sonicated *P. gingivalis* induced inflammatory response of MEPM cells **(a)** qPCR assay of gene encoding inflammatory cytokine (n=3). **(b)** The production of inflammatory cytokine measured by using ELISA kits (n=3). The data are shown as the mean ±SD and were statistically analysed by two-tailed Student’s t-test.

**Supplemental Tables**

Supplementary Table 1 Frequency of cleft palate in embryos under *P. gingivalis* treatment

| Group |  | | Control | | | |  | *P. gingivalis* | |
| --- | --- | --- | --- | --- | --- | --- | --- | --- | --- |
|  | Pregnant mice | | cleft palate/Total embryos | | Frequency of cleft palate | Pregnant mice | | cleft palate/Total embryos | Frequency of cleft palate |
| E16.5 in the 1^st^ round of experiment | 6 | | 0/38 | | 0 | | 6 | 4/33 | 12.1% |
| E15.5 in the 1^st^ round of experiment | 6 | | 0/28 | | 0 | | 6 | 3/23 | 13.0% |
| E16.5 in the 2^nd^ round of experiment | 6 | | 0/40 | | 0 | | 6 | 4/32 | 12.5 % |
| Total cleft palate/Total embryos | |  | | 0/106 | 0 | |  | 11/88 | 12.5% |

Supplementary Table 2 The number of fetuses with CP born from each mother

| Group | Control | | | *P. gingivalis* | | |
| --- | --- | --- | --- | --- | --- | --- |
| E16.5 in the 1^st^ round of experiment | 0/6 | 0/7 | 0/6 | 1/8 | 1/5 | 1/6 |
|  | 0/5 | 0/8 | 0/6 | 0/5 | 0/5 | 1/4 |
| E15.5 in the 1^st^ round of experiment | 0/4 | 0/7 | 0/2 | 1/4 | 1/6 | 0/4 |
|  | 0/5 | 0/6 | 0/4 | 1/5 | 0/1 | 0/3 |
| E16.5 in the 2^nd^ round of experiment | 0/8 | 0/5 | 0/7 | 0/5 | 1/5 | 2/7 |
|  | 0/5 | 0/7 | 0/8 | 1/6 | 0/4 | 0/5 |

Supplementary Table 3 The antibodies used for western blot

| Antibodies | Source | Identifier |
| --- | --- | --- |
| TGFBR1 | Abcam | ab235578 |
| p-SMAD2 | Cell Signaling Technology | 3108S |
| p-SMAD3 | Cell Signaling Technology | 9520S |
| SMAD2/3 | Cell Signaling Technology | 8685S |
| HK2 | Proteintech | 22029- 1-AP |
| LDHA | Proteintech | 19987-1-AP |
| CytC | Abclonal | A13291 |
| H3K18la | PTM Bio | PTM-1406RM |
| H4K5la | PTM Bio | PTM-1409 |
| H4K12la | PTM Bio | PTM-1411 |
| H3 | Abclonal | a2348 |
| Pan kla | PTM Bio | PTM-1401 |
| H4 | PTM Bio | PTM-1009 |
| ADAM17 | Proteintech | 299948-1-AP |
| MerTK | Abcam | ab300136 |
| CD206 | Proteintech | 18704-1-AP |
| CD86 | Proteintech | 26903-1-AP |
| CD63 | Proteintech | 67605-1-lg |
| TSG101 | Proteintech | 28283-1-AP |
| BMP2 | Abclonal | A14708 |
| OPN | Abclonal | A19092 |
| Runx2 | Abclonal | A11753 |
| α-Tubulin | Abclonal | AC007 |
| β-Actin | Proteintech | 66009-I-IG |
| GAPDH | Proteintech | 60004-I-IG |

Supplementary Table 4 The primer sequences used for qRT-PCR and ChIP-qPCR

| Primers | Sequences |
| --- | --- |
| *Runx2*-F | CCGCCTCAGTGATTTAGGGC |
| *Runx2*-R | GGGTCTGTAATCTGACTCTGTCC |
| *Osx*-F | CTGGCTAGGTGGTGGTCAG |
| *Osx*-R | GTAGGGAGCTGGGTTAAGG |
| *Ocn*-F | GGGCAATAAGGTAGTGAACA |
| *Ocn*-R | GTCTTCAAGCCATACTGGTC |
| *Alp*-F | CCAACTCTTTTGTGCCAGAGA |
| *Alp*-R | GGCTACATTGGTGTTGAGCTTTT |
| *TNF-α*-F | GACAAGGCTGCCCCGACTACG |
| *TNF-α*-R | CTTGGGGCAGGGGCTCTTGAC |
| *IL-6*-F | TGATGGATGCTACCAAACTGGA |
| *IL-6*-R | GTGACTCCAGCTTATCTCTTGGT |
| *IL-1β*-F | TGCCACCTTTTGACAGTGATG |
| *IL-1β*-R | TGTGCTGCTGCGAGATTTGA |
| *Adam17*-F (ChIP-qPCR) | TCCATCTCCCAGCAGACTCA |
| *Adam17*-R (ChIP-qPCR) | AGAGGGCGCAGAACACTAAC |
| *β-actin*-F | GTGACGTTGACATCCGTAAAGA |
| *β-actin*-R | GCCGGACTCATCGTACTCC |
